# Molecular Targets and Biological Functions of cAMP Signaling in *Arabidopsis*

**DOI:** 10.3390/biom11050688

**Published:** 2021-05-03

**Authors:** Ruqiang Xu, Yanhui Guo, Song Peng, Jinrui Liu, Panyu Li, Wenjing Jia, Junheng Zhao

**Affiliations:** 1 School of Agricultural Sciences, Zhengzhou University, Zhengzhou 450001, China; omapeh01@gmail.com (Y.G.); pengsong9509@gmail.com (S.P.); ljr111666@gmail.com (J.L.); lipy0405@gmail.com (P.L.); wj210403@gmail.com (W.J.); zhaojh9605@gmail.com (J.Z.); 2 Zhengzhou Research Base, State Key Laboratory of Cotton Biology, Zhengzhou University, Zhengzhou 450001, China

**Keywords:** *Arabidopsis thaliana*, cyclic AMP, signaling, mechanism, function, regulation, adenylate cyclase

## Abstract

Cyclic AMP (cAMP) is a pivotal signaling molecule existing in almost all living organisms. However, the mechanism of cAMP signaling in plants remains very poorly understood. Here, we employ the engineered activity of soluble adenylate cyclase to induce cellular cAMP elevation in *Arabidopsis thaliana* plants and identify 427 cAMP-responsive genes (CRGs) through RNA-seq analysis. Induction of cellular cAMP elevation inhibits seed germination, disturbs phytohormone contents, promotes leaf senescence, impairs ethylene response, and compromises salt stress tolerance and pathogen resistance. A set of 62 transcription factors are among the CRGs, supporting a prominent role of cAMP in transcriptional regulation. The CRGs are significantly overrepresented in the pathways of plant hormone signal transduction, MAPK signaling, and diterpenoid biosynthesis, but they are also implicated in lipid, sugar, K^+^, nitrate signaling, and beyond. Our results provide a basic framework of cAMP signaling for the community to explore. The regulatory roles of cAMP signaling in plant plasticity are discussed.

## 1. Introduction

Cyclic AMP (cyclic 3′,5′-adenosine monophosphate; cAMP) is a second messenger molecule present in almost all living organisms, which plays a pivotal role in cell signaling and modulates a variety of cellular responses. cAMP was first discovered to mediate the effects of hormones in mammalian cells [1] and later demonstrated to regulate signaling pathways critical for adaptation and survival in many lower eukaryotes [2,3,4] and modulate gene expression involving antibiotic production, phototrophic growth, pathogenesis and nitrogen fixation in prokaryotes [5]. However, the existence and functions of cAMP were controversially studied for a period of about thirty years in higher plants [6,7,8,9,10,11]. While cAMP is currently taken as an important component of the complex signaling network in plants [12,13,14], the underlying molecular mechanism remains largely unknown, especially regarding the molecular targets and biological pathways.

Cellular cAMP levels were generally much lower in higher plants than animals [9,11]. However, recent advances from animal studies suggest that cAMP signaling may occur in subcellular compartment or at microdomains near the sites of cAMP production for the fidelity and efficiency of signal transduction in response to specific stimuli [15,16]. cAMP is universally produced from ATP by adenylate cyclase (AC) [17], whereas phosphodiesterase (PDE) catalyzes its hydrolytic degradation to form AMP [18]. Although cellular levels of cAMP principally reflect a balance between its synthesis and degradation, cAMP signaling events are largely dependent on the activation of ACs to rapidly increase cAMP, which stimulates downstream effectors, such as cyclic nucleotide gated channels (CNGCs) and protein kinase A (PKA); in contrast, PDEs are responsible for bringing the concentration of cAMP back to the basal level for the cell responding to a new stimulus [19,20]. This notion was supported by a recent study showing few differential expression proteins (DEPs) identified between the cAMP-deficiency transgenic plants and wild type plants [21]. All known ACs in eukaryotes, along with members from many prokaryotes, have been phylogenetically grouped into the ubiquitous Class III that are defined by a highly conserved ~200 residue catalytic domain [17,22]. In animals, both forms of membrane-bound AC (mAC) and soluble AC (sAC) are present, and they are differentiated by the former containing transmembrane domains [22]. While the mACs are mainly regulated via G protein-coupled receptors (GPCRs) and heterotrimeric G proteins, and the sAC is directly activated by the intracellular signaling molecules bicarbonate (HCO_3_^−^) and calcium, they are also unraveled with many unifying themes for functions in cellular signaling [17]. It is believed that the sAC may represent an ancient and fundamental regulatory component per the evolutionary conservation of bicarbonate regulation [10].

Plant tissue extracts displayed both mAC and sAC activities, and these activities were present across various cellular organelles including the nucleus, chloroplasts, mitochondria, and vacuoles [23,24,25,26,27,28,29,30]. Comparable sAC activities were observed between plants and animals, and the activities of both mAC and sAC in plants were significantly impacted by external factors, such as far-red and red light, temperature, and exogenous phytohormones, as well as specific triggering compounds of fungal and bacterial origin [23]. Unfortunately, only a few genes that code for proteins having AC activities were identified in plants until now. Of them, *PSiP* from maize (*Zea Mays*), is a pollen-specific gene essential for pollen tube growth and encodes a homolog of nucleotide binding site-leucine-rich repeat (NBS-LRR) disease resistance proteins in *Arabidopsis* [31]; *HpAC1* from *Hippeastrum x hybridum* was identified to involve mechanical damage and fungal infection [32], and it encodes an orthologue of *Arabidopsis* AtTTM3, a tripolyphosphatase without any AC activity [33]; *NbAC* from *Nicotiana benthamiana* was implicated in tabtoxinine-β-lactam-induced cell death and wildfire disease development [34]. BLAST homology searches based on annotated cyclic nucleotide cyclases (CNCs) from other living organisms failed to identify genes encoding either ACs or guanylyl cyclases (GCs) in plant genomes, whereas determination of functionally conserved amino acids forming the catalytic center of plant-origin ACs using bioinformatic analysis has successfully identified several genes possessing AC activities recently [11,35,36,37]. These genes each feature a distinctive function different from its AC activity, including AtKUP5 and AtKUP7, which are both essential for K^+^ transport in plants [38,39], AtLRRAC1 being the LRR protein implicated in immune responses [36], AtCIAP annotated with a role in clathrin assembly and endocytosis [40], and the pentatricopeptide repeat-containing protein AtPPR-AC [41]. It has recently been suggested that plant genomes encode numerous complex multidomain proteins, which have well-documented roles in development and responses to the environment, containing the functional AC centers [35]. Obviously, it may be necessary to employ a genetic model solely expressing AC enzyme activity, just like that in animals, for the systemic dissection of cAMP signaling in plants.

Biological effects of cAMP in plants were previously investigated mostly depending on the pharmacological approach using exogenous cAMP, AC activators, and inhibitors. cAMP was found to impact hormonal actions [42,43,44,45,46], phytochrome responses [47,48], cell cycle [46,49,50,51], stomatal opening [52], phytoalexin biosynthesis [53,54], pollen tube growth [31], and ion transport and homeostasis [38,55]. The stimulation of transient cAMP elevation was observed in plant responses to anoxia [56], wounding [57], salt [58], heat [59], H_2_O_2_ [60], and pathogens [21,61]. In addition, cellular cAMP levels in plants were affected by the environmental conditions of temperature and light [48,59,62]. While all these findings collectively support diverse roles of cAMP in plant development and environmental responses, the underlying molecular mechanisms are rarely known.

In recent years, efforts have been made to address the molecular mechanism of cAMP signaling in plants. Using exogenous cAMP application, proteomics analysis revealed 76 DEPs from *Arabidopsis* seedlings, which are implicated in light- and temperature-dependent responses [63]; additionally, twenty DEPs were determined from *Arabidopsis* cell suspension culture, which function in biotic and abiotic stress responses, and glycolysis [64]. More recently, the so called “cAMP sponge” (cAS) that contains the C-terminus of human PKA regulatory Iβ subunit was used to reduce endogenous cAMP availability by genetic transformation in plants. Consequently, it was found that cAMP-deficiency in tobacco BY-2 cells inhibited cell growth and enhanced stress-related responses, resulting in the identification of 94 DEPs, which are functionally related to translation, cytoskeletal organization, and cell proliferation and stress responses, as well as ubiquitin/proteasome-mediated protein degradation [50]; in contrast, only four DEPs were revealed between the cAS transgenic *Arabidopsis* plants and wild type plants at resting conditions, and 18 DEPs were obtained by bacterial infection, which are functionally implicated in transport, photosynthesis, redox control, translation, and metabolism [21]. Over recent years, it has become increasingly evident that cAMP regulates the functions of CNGCs in plants [55,65,66,67]. During the perception of pathogens in *Arabidopsis*, cAMP elevation was linked to AtCNGC2 activation, which in turn caused a cytosolic Ca^2+^ increase, triggering downstream nitric oxide (NO) and reactive oxygen species (ROS) generation, as well as immune signaling, leading to a hypersensitive response [61]. cAMP was suggested to act upstream of salicylic acid (SA) during the plant defense process and induced *PR1* expression [68], and it modulated jasmonic acid (JA)-mediated signaling pathway [69]. Interestingly, twelve cyclic nucleotide binding proteins were isolated by affinity purification of *Arabidopsis* protein extracts, which included key enzymes in the Calvin cycle and photorespiration pathway [70]. These proteins are post-translationally modified by NO, transcriptionally co-expressed, and annotated to function in H_2_O_2_ signaling and the defense response, and thus have been suggested to act together as points of crosstalk between cyclic nucleotide, NO, and ROS signaling during the defense response. Altogether, these advances are very limited and represent the very beginning of elucidating cAMP signaling cascades in plants, which require comprehensive and systemic studies.

## 2. Materials and Methods

### 2.1. Transgenic Plants and Growth Conditions

The cDNA fragment (312 bp) encoding the N-terminal cytosolic region of AtKUP7 in *Arabidopsis thaliana* was PCR-amplified with the addition of stop codon (TAA) using the template of pDEST-AtKUP7^1−100^ (kindly provided by Prof. Chris Gehring, King Abdullah University of Science and Technology, Thuwal, Saudi Arabia). The PCR product was subcloned into a Gateway^@^-compatible pTA7001 vector [71], resulting in pTA7001-AC. Using *Agrobacterium tumefaciens* GV3101, pTA7001-AC was introduced into *Arabidopsis thaliana* wild type Col-0 plants by the floral dip method as described previously [72]. Transgenic plants homozygous for a single copy T-DNA insertion in the genome were screened by segregation of hygromycin resistance in the progenies. Plants were grown on potting mix (Pindstrup Mosebrug A/S, Denmark) in a growth room of 16 h light/8 h dark and 22 °C.

### 2.2. DEX Treatment

Dexamethasone (DEX, Sigma-Aldrich) was dissolved in DMSO for a stock solution of 30 mM and used in dilution [71]. Plants grown in soil were thoroughly sprayed with a water solution containing 30 µM DEX with the addition of 0.01% (*w*/*v*) Tween-20 as a wetting agent, and then covered overnight using a transparent plastic dome. Seedlings grown in agar medium were treated by the addition of DEX at a final concentration of 10 µM. For the mock control, the same amount of DMSO and/or Tween-20 was used.

### 2.3. cAMP Extraction and Measurement

Extraction and measurement of cAMP in plant tissue samples were performed following a previous publication [61]. Briefly, fresh tissues were collected and immediately ground in liquid nitrogen. Tissue powder was extracted with the addition of 1 M HClO_4_ and then neutralized with KOH. The extract was clarified by centrifugation. Supernatants were lyophilized for cAMP quantification using a commercial cAMP ELISA Detection Kit (GenScript, Jiangsu, China).

### 2.4. RNA Extraction, cDNA Library Construction, and Transcriptome Sequencing

Six-week-old pTA7001-AC transgenic plants were randomly separated into two groups and then thoroughly sprayed with DEX solution as described above. Whole plant tissue samples were collected immediately (0 h) and 24 h after spraying, each with three biological repeats. Total RNA was isolated using TRIzol reagent (Invitrogen). RNA concentration, purity, and integrity were examined using NanoDrop™ 2000 spectrophotometer (Thermo Fisher Scientific), Agilent Bioanalyzer 2100 (Agilent Technologies), and agarose gel electrophoresis. Sequencing libraries were prepared using NEBNext^®^ Ultra™ RNA Library Prep Kit for Illumina^®^ (New England Biolabs) following the manufacturer’s recommendations. The average insert size for the final cDNA library was ~350 bp. Library quality was assessed on the Agilent Bioanalyzer 2100 system and quantified by Qubit 2.0 Fluorometer (Invitrogen). The library preparations were sequenced on an Illumina sequencing platform with paired-end (2 × 150 bp) reads (Origin-gene Bio-pharm, Shanghai, China). Raw reads were cleaned by removing adapter sequences and trimming the ends of low-quality and unknown bases (N) using cutadapt v1.16 software (http://cutadapt.readthedocs.io (accessed on 14 November 2019)). Sequence quality was verified using FastQC (http://www.bioinformatics.babraham.ac.uk/projects/fastqc/ (accessed on 17 November 2019)).

### 2.5. Transcripts Assembly, Annotation, and Quantification

RNA-seq clean reads were aligned to *Arabidopsis thaliana* reference genome (Ensembl, version TAIR10.45; ftp://ftp.ensemblgenomes.org/pub/plants/release-45/fasta/arabidopsis_thaliana/ (accessed on 18 November 2019)) using the HISAT2 v2.1.0 program (https://daehwankimlab.github.io/hisat2/ (accessed on 18 November 2019)) and allowing two base mismatches. The quality of RNA-seq experiment was assessed using RSeQC v2.6.4 (http://rseqc.sourceforge.net/ (accessed on 21 November 2019)). The mapped reads were assembled using StringTie v1.3.3b (http://ccb.jhu.edu/software/stringtie/ (accessed on 22 November 2019)). All assembled transcripts were annotated by BlastX search against public databases. Read counts for each gene model were obtained using the StringTie, and gene expression level was estimated using FPKM (fragments per kilobase of exon model per million mapped fragments). The DEGs (differentially expressed genes) were selected with an absolute value of log2 (fold change) >1 and a false discovery rate (FDR) adjusted *p*-value < 0.05 using the edgeR v3.24 (http://www.bioconductor.org/packages/release/bioc/html/edgeR.html (accessed on 27 November 2019)).

### 2.6. Bioinformatics Analysis

PCA was performed using ClustVis [73]. A Venn diagram was generated using an online tool (http://bioinformatics.psb.ugent.be/webtools/Venn/ (accessed on 23 June 2020)). Unless noted otherwise, functional annotation enrichment analyses were conducted using DAVID v6.8 (https://david.ncifcrf.gov/ (accessed on 4 August 2020)) with the embedded resource of GO DIRECT that provides GO mappings directly annotated by the source database (no parent terms included), and the statistical significance was determined by the cutoff of Benjamini–Hochberg adjusted FDR < 0.05. A network-based visualization for functional enrichment results was performed using the EnrichmentMap (http://baderlab.org/Software/EnrichmentMap (accessed on 6 August 2020)) plugin of Cytoscape v3.8.0 (https://cytoscape.org/ (accessed on 6 August 2020)). The over-representation analyses of TF families in the CRGs were based on PlantTFDB v5.0 (http://planttfdb.cbi.pku.edu.cn/ (accessed on 24 July 2020)), and a two-sided Fisher’s exact test was performed to determine statistical significance by a *p*-value < 0.05 using GraphPad Prisms 8.0.2 software (https://www.graphpad.com/ (accessed on 25 July 2020)).

### 2.7. GSEA

The cAMP-responsive gene sets were determined using GSEA (Gene Set Enrichment Analysis) [74]. Briefly, the RNA-seq expression data set was ranked from the highest to the lowest by the metric log2(fold change), and then used as inputs to the GSEA software with the default options. The pre-defined gene sets were based on GO terms and KEGG pathways, which were filtered in size (min = 10, max = 500) after excluding any gene not in the expression data set. An enrichment score (ES) for each gene set was calculated, and the normalized enrichment scores (NES) were used to compare the analysis results across gene sets. Per the recommendation of GSEA, a gene set was considered significantly enriched if its NES has an FDR < 0.25, especially for a large size of set (rule of thumb: more than 30). RANK AT MAX is the position in the ranked list at which the maximum enrichment score occurred. The leading-edge subset was extracted to represent the core members of each gene set that contribute most to the ES. Three statistics were used to define the leading-edge subset: tags, the percentage of gene hits before (for positive ES) or after (for negative ES) the peak in the running ES; list, the percentage of genes in the ranked gene list before or after the peak in the running ES; and signal, the enrichment signal strength that combines the two previous statistics.

### 2.8. WGCNA and Network Visualization

Co-expression network analysis was performed using WGCNA (Weighted Correlation Network Analysis) software [75]. Briefly, the RNA-seq data set of gene expression was pre-processed by filtering out the genes with little variation or unusual expression, and then log-transformed FPKM values were used to create a matrix of pairwise Pearson correlations between all pairs of genes across the measured samples. This matrix was transformed into a signed adjacency matrix and analyzed to make the whole network fit the scale-free topology using a soft-threshold power function. Based on the adjacency matrix, the topological overlap (TO) was calculated, which is a measure of network interconnectedness reflecting the strength of two genes’ co-expression relationship with respect to all other genes in the network. Genes with highly similar co-expression relationships were grouped together by performing average linkage hierarchical clustering on the TO. The Dynamic Hybrid Tree Cut algorithm was used to cut the hierarchal clustering tree, and modules were determined as branches from the tree cutting. The module eigengene (ME) was estimated to represent the expression profile of each module by the first principal component, and modules whose eigengenes were highly correlated (correlation above 0.7) were merged. The MEs were correlated to the traits using the Pearson correlation. A gene’s module membership (MM), also known as eigengene-based connectivity K_ME_, was calculated as the Pearson correlation between the gene’s expression and ME, which is highly related to the intramodular connectivity (sum of the weight of intramodular edges or sum of connection weights with all other nodes). Thus, highly connected intramodular genes tend to have high-ranking K_ME_ within the module. For an easy visualization of networks, members in each module were ranked by their K_ME_ values and the top 10% highest ranking members were selected for the network depiction graphically using Cytoscape [76]. Thus, these “network depictions” show only the strongest reciprocal within-module gene–gene interactions (“connections”) as measured by TO.

### 2.9. Phytohormone Quantification

Plant tissue samples were accurately weighted, homogenized, and extracted with methanol by incubation overnight. After repeated extraction with vortex mixing and centrifugation, the supernatants were pooled and subjected to purification using a pre-equilibrated solid phase extraction (SPE) column. The column was briefly washed with 15% methanol prior to elution with 5 mL methanol. The eluent was evaporated to dryness with nitrogen, and the residue was dissolved in 1 mL of 20% methanol. After centrifugation, supernatants were collected and loaded onto an ultra-performance liquid chromatography triple quadrupole tandem mass spectrometer for analyses in multiple reaction monitoring (MRM) mode using the AB SCIEX Q-TRAP^®^ 6500 LC-MS/MS system (Applied Biosystems). Each sample was analyzed in triplicates. A standard curve was prepared using a serial dilution of the analytical phytohormone standards by plotting the concentration (*x*-axis) and the obtained peak area (*y*-axis), which was used to determine the concentration of salicylic acid (SA), jasmonate (JA), abscisic acid (ABA), kinetin (6-KT), gibberellic acid (GA_3_), gibberellin 4 (GA_4_), and indole-3-acetic acid (IAA) in the samples. All data were processed using the AB Analyst^®^ software (Applied Biosystems).

### 2.10. Phenotypic Characterization

For the germination test, seeds were sown on wet filter paper that was placed on the surface of 1/2 MS medium with or without the addition of 10 µM DEX. Radicle protrusion was the criterion for germination. The germinated seeds were counted for a period of seven days, and the seed germination percentage or rate each day were determined. For the bacterial infection test, plants were grown for four to five weeks on potting mix covered with fine mesh veil, and *P. syringae* pv *tomato* DC3000 (*Pst* DC3000) was cultured in LB medium at 28 °C and prepared in suspension with a final OD_600_ of 0.5 in a water solution containing 10 mM MgCl_2_ and 0.02% of the surfactant Silwet L-77. After 24 h of pre-treatment by spraying DEX or the mock control as described above, plants were inoculated by submerging inverted pots in the bacterial suspension for 3 min until all the leaves appeared water-soaked, and then laid down overnight by covering a transparent dome; leaf disk samples were collected 10 days post-inoculation, homogenized with sterile water, and cultured on LB plates containing 20 µg/mL rifampicin for the determination of colony-forming unit (cfu). For the fungal test, *V. dahlia* strain Vd991 was cultured with Czapek medium at 25 °C for 3–5 days and then filtered through four layers of sterile gauze for obtaining conidial suspension; fully expanded rosette leaves with the petiole were detached from 34-day-old plants, scraped to give a 2 mm long wound on the basal midrib surface of abaxial leaf using a syringe needle, followed by a drop of 2 µL conidial suspension (1 × 10^8^ spores/mL) on the wound, and then the petiole was inserted into a water solution containing 10 µM DEX or mock control in an Eppendorf tube; after 10 days of incubation at room temperature, genomic DNA was extracted from the inoculated leaves and used as template for real-time PCR analysis using specific primers of Vd991 (Supplementary Table S1) that were designed to target the internal transcribed spacer region (ITS) of the 5.8 S ribosomal RNA gene; the *Arabidopsis thaliana* Actin (*AtACT2*) gene was used for sample equilibration. The relative biomass of Vd991 was measured as a ratio of fungus to plant DNA [77], which was calculated using the 2^−ΔΔCq^ method. For testing plant responses to abiotic stresses and phytohormones, 12-day-old seedlings were prepared by growing on 1/2 MS basal salt medium (containing 1% sucrose) and then transplanted onto the growth medium with the addition of 10 µM DEX or mock control in combination with various tested conditions as indicated in the context.

### 2.11. Quantitative RT-PCR

Total RNA was isolated using the Spin Column Plant Total RNA Purification Kit (Sangon Biotech, Shanghai, China). After DNase I digestion, total RNA was used for reverse transcription (RT) using the HIScript^®^ III RT SuperMix for qPCR (+ gDNA wiper) reagent kit (Vazyme Biotech, Jiangsu, China). PCR reaction mixture was prepared using the ChamQ^TM^ Universal SYBR^®^ qPCR Master Mix (Vazyme Biotech) and then loaded onto LightCycler^®^ 480 II Real-Time PCR System (Roche) for amplification with the following conditions: 30 s at 95 °C, 40 cycles of 95 °C for 10 s, and 60 °C for 30 s. The specificity of amplified products was monitored by melting curve analysis and verified by agarose gel electrophoresis. Gene-specific primers used in this study were listed in Supplementary Table S1. β-tubulin (*AtTUB4*) was used as an internal control to calculate the relative expression levels of target genes using the 2^−ΔΔCq^ method.

## 3. Results

### 3.1. Establishment of an Inducible System for Adenylate Cyclase Activity and cAMP Elevation in Plants

**Figure 1:**
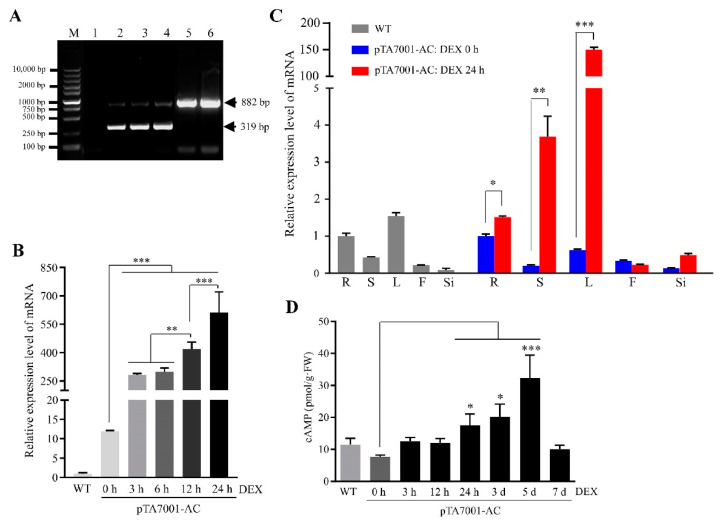
Generation of inducible *AC* transgenic plants mediating cAMP elevation. (**A**) Verification of *AC* transgenic plants. The pTA7001-AC construct was transformed into *Arabidopsis thaliana* wild type (WT) Col-0 background to obtain transgenic plants homozygous for a single copy of *AC* transgene. PCR products from genomic DNA samples of three individual transgenic lines (lanes 2–4) showed a band of 319 bp corresponding to the *AC* transgene and a band of 882 bp corresponding to *AtKUP7*, and only the band of 882 bp appeared in WT plants (lanes 5–6) and no amplification in the non-template control (lane 1). (**B**) Inducible expression of *AC* transgene examined by qRT-PCR in rosette leave samples of four-week-old WT and *AC* transgenic plants at different times (0, 3, 6, 12, and 24 h) after spraying dexamethasone (DEX). (**C**) Inducible expression of *AC* transgene examined by qRT-PCR in different tissue (R: roots; S: stems; L: rosette leaves; F: flowers; Si: siliques) samples of six-week-old WT and *AC* transgenic plants at 0 and 24 h after spraying DEX. (**D**) Inducible elevation of cellular cAMP contents in the above-ground tissue samples of three-week-old WT and *AC* transgenic plants at different times (0 h, 3 h, 12 h, 24 h, 3 d, 5 d, and 7 d) after spraying DEX. Data are Ave ± SD (*n* = 3) in (**B**,**C**), and Ave ± SE (*n* = 3) in (D), * *p* < 0.05, ** *p* < 0.01, and *** *p* < 0.001 with Fisher’s LSD test following ANOVA.

The N-terminal cytosolic region of *Arabidopsis* AtKUP7 (At5g09400) contains an adenylate cyclase catalytic center [39], and its corresponding cDNA fragment with the addition of stop codon (TAA) was introduced into pTA7001, a glucocorticoid-mediated transcriptional induction system [71]. Transgenic plants homozygous for a single copy of T-DNA insertion were obtained with *Arabidopsis thaliana* wild type (WT) Col-0 background. The transgene was referred to as *AC* hereafter. Using genomic DNA as a template and *AC* gene-specific primers (Supplementary Table S1) to conduct PCR, we confirmed *AC* transgenic lines, which showed two DNA bands corresponding to an expected size (319 bp) of the *AC* transgene and a partial DNA fragment (882 bp) of the native *AtKUP7*, whereas only the 882 bp band was detected in WT plants (Figure 1A). To induce *AC* expression, transgenic plants were thoroughly sprayed with a water solution containing 30 µM dexamethasone (DEX, a strong synthetic glucocorticoid) with the addition of 0.01% (*w*/*v*) Tween-20 as a wetting agent [71,72]. Given the concern that the spraying compounds may potentially stimulate native adenylate cyclase activities in WT plants, we collected tissue samples immediately (0 h) after spraying DEX in *AC* transgenic plants to serve as the controls, which rendered us to pinpoint the effects of induced cAMP elevation by comparison under the regime of identical genetic background using *AC* transgenic plants in this study.

Leaf samples of four-week-old *AC* transgenic plants were collected during a time course after spraying DEX and detected for *AC* expression by quantitative RT-PCR (qRT-PCR), indicating that *AC* transcripts increased by 23.6-, 25.1-, 35.1-, and 51.3-fold at 3, 6, 12, and 24 h compared to 0 h (Figure 1B). While both the transcripts of *AC* and *AtKUP7* were simultaneously detected by the designed primers, it was noted that the transcript levels of 0 h leaf sample from *AC* transgenic plants were apparently higher than WT plants grown in parallel (Figure 1B), suggesting the leaky expression of transgene commonly observed with chemical-inducible systems [72,78,79]. We detected and compared expression patterns of *AC* and *AtKUP7* in different tissues collected from six-week-old plants. Comparison of *AC* transgenic plant tissue samples of 24 versus 0 h after spraying DEX indicated that the transcript levels increased by 1.5-, 18.5-, and 242.0-fold in roots (R), stems (S), and rosette leaves (L), respectively (Figure 1C). Analysis of variance (ANOVA; F (9, 20), *p* < 0.0001) followed by Fisher’s LSD multiple comparisons indicated significant expression changes in the above-mentioned tissues, but not in flowers (F) and siliques (Si); however, expression changes in all these tissues were significant as determined by Student’s *t*-test. In comparison, expression levels of *AtKUP7* were highest in rosette leaves, followed by the order in roots, stems, flowers, and siliques in WT plants (Figure 1C).

To confirm an inducible activity of adenylate cyclase to synthesize cAMP in *AC* transgenic plants, we collected above-ground tissues of three-week-old seedlings during a time course after spraying DEX and measured cellular cAMP levels. The results indicated that cAMP increased from 7.59 pmol/g·FW (fresh weight) at 0 h to 12.62 pmol/g·FW at 3 h, then slightly reduced to 12.00 pmol/g·FW at 12 h and bounced back to continually increase to 17.56 pmol/g·FW at 24 h and 20.18 pmol/g·FW at 3 d, and finally reached a peak of 32.30 pmol/g·FW at 5 d and subsequently declined to 10.08 pmol/g·FW at 7 d after spraying (Figure 1D). ANOVA (F (7,16), *p* = 0.002) followed by Fisher’s LSD multiple comparisons indicated significant elevation of cAMP at 24 h, 3 d, and 5 d versus 0 h after spraying DEX. It was noted that the duration of the induced AC activities conformed very well to previously characterized features of the inducible system in planta [71,80].

Taken together, the above results confirmed the rapid induction of *AC* expression and concomitantly elevated cAMP levels in the transgenic plants. Importantly, the induced accumulation of cAMP seemed to exhibit a biphasic profile (Figure 1D, Student’s *t*-test *p* = 0.0274 between 3 h vs. 0 h, *p* = 0.0653 between 12 h vs. 0 h), which is in line with the well-known biphasic regulation of cAMP production in animals [81,82], and similar to the accumulation of cGMP in pathogen-challenged plants that is usually required for the perception and responses of such a transient signal in nature [83]. Therefore, our *AC* transgenic plants may serve as an ideal genetic model for studying cAMP signaling. A recent report indicated that the stimulated elevation of cAMP occurred at 4 h and increased further at 24 h after pathogen infection in *Arabidopsis* WT plants [21]. Thus, we conducted systemic dissection of cAMP signaling per the regime of 24 h after spraying DEX in *AC* transgenic plants.

### 3.2. Global Profile of Gene Expression Changes Associated with Cellular cAMP Elevation in plants

**Figure 2:**
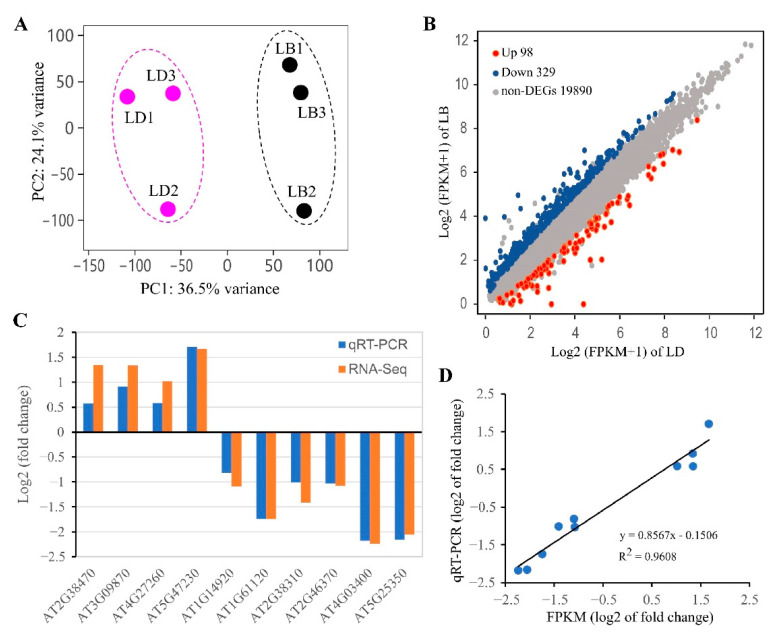
Identification of cAMP-responsive genes (CRGs). (**A**) Principal component analysis (PCA) of transcriptome changes associated with induction of cAMP elevation using FPKMs data derived from RNA-seq of six-week-old *AC* transgenic plants at 24 h (labeled as LD) and 0 h (LB) after spraying dexamethasone (DEX), each with three biological replicates. (**B**) Scatter plot showing the relationship between magnitude of gene expression in a comparison of LD and LB. CRGs (Supplementary Data S1) were identified by differentially expressed genes (DEGs) at the threshold of an absolute value of log2 (fold change) > 1 and a false discovery rate (FDR) < 0.05. (**C**) Verification of RNA-seq data. Relative expression levels of 10 randomly selected CRGs by qRT-PCR were compared to their FPKMs by RNA-seq. (**D**) Correlation between the results of qRT-PCR and RNA-seq as shown in (**C**).

The *AC* transgenic plants were cultivated for six weeks to early fruiting stage, and then whole plant tissue samples with three biological replicates were collected at 24 h (labeled as LD) and 0 h (LB) after spraying DEX. RNA-seq analyses were performed using total RNA, and high-quality clean reads were generated with an averaged Q20 of 97.00% (SD = 0.17%) and Q30 of 91.90% (SD = 0.34%) across all the sequencing libraries. An average of 21,070,368 clean reads per library were mapped to *Arabidopsis thaliana* reference genome, resulting in an averaged mapping rate of 91.13% (SD = 0.88%) and unique mapping rate 88.07% (SD = 0.72%). Finally, a total of 28,010 expressed genes were determined by the assembly step, accounting for 83.36% of the total annotated genes (33,602) in the genome (TAIR10) [84]. In addition, 149 and 132 genes from the mitochondria and chloroplast genomes were detected, respectively. FPKM (fragments per kilobase of exon model per million mapped fragments) was estimated for each gene, and principal component analysis (PCA) confirmed two distinct clusters of LD versus LB samples, with the PC1 and PC2 together accounting for 60.6% of the expression variance (Figure 2A). Comparison of LD versus LB identified 427 differentially expressed genes (DEGs) at the threshold of an absolute value of log2(fold change) > 1 and a false discovery rate (FDR) < 0.05 (Figure 2B; Supplementary Data S1). These DEGs were determined as cAMP-responsive genes (CRGs), which included 329 down-regulated CRGs (down-CRGs) and 98 up-regulated CRGs (up-CRGs). The reliability of RNA-seq data was validated by qRT-PCR detection of 10 randomly selected up- and down-CRGs using gene-specific primers (Supplementary Table S1), showing a highly significant correlation (R^2^ = 0.96, *p* < 0.001) between the results of RNA-seq and qRT-PCR (Figure 2C,D). Additionally, we examined relative expression of CRGs in independent AC transgenic lines and WT plants under conditions of DEX treatment and mock control, and the results showed the induced expression of CRGs in the *AC* transgenic lines, but not in WT plants, confirming the specific effects of DEX treatment or cAMP elevation in the transgenic plants (Supplementary Figure S1).

**Figure 3:**
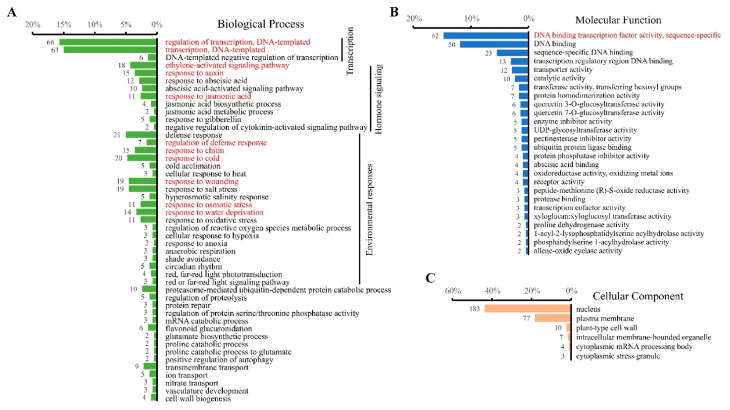
Functional characteristics of cAMP-responsive genes (CRGs). (**A**) Bar plot showing the functional terms of Biological Process by Gene Ontology (GO; Supplementary Data S2) analysis with CRGs (Supplementary Data S1). (**B**) Bar plot showing the functional terms of molecular function by GO analysis with CRGs. (**C**) Bar plot showing the functional terms of the cellular component by GO analysis with CRGs. In (**A**) to (**C**), GO terms in red indicate significant enrichment at the false discovery rate (FDR) < 0.05. Shown are the number of CRGs associated with the given GO term on the top of each bar and its percentage among the CRGs on the top of each bar plot.

All the CRGs were subjected to Gene Ontology (GO)-based functional annotation analyses using DAVID [85]. The results indicated the association of CRGs with 79 GO terms, including 47 from the category of Biological Process (BP), 26 from the Molecular Function (MF), and six from the Cellular Component (CC) (Figure 3A–C; Supplementary Data S2). Per the cutoff of FDR < 0.05, the significantly enriched BP terms are related to transcription (regulation of transcription), ethylene-activated signaling pathway, responses to phytohormones (auxin, JA), defense (regulation of defense response, response to chitin), and responses to abiotic stress (cold, wounding, osmotic, and water deprivation). These results suggested the functions of cAMP elevation in significant relevance to transcription, hormone signaling, and biotic and abiotic stress responses. However, a dominant number of CRGs were functionally related to transcription (Figure 3A), suggesting a prominent role of cAMP elevation associated with transcription. Consistently, DNA binding transcription factor activity was the only significantly enriched term of MF (Figure 3B). While no CC terms were significantly enriched, a great number of CRGs were associated with the nucleus, followed by plasma membrane (Figure 3C). It is worth pointing out that all other GO terms that were not significant by the determination of arbitrarily set criteria should not be neglected for their relevance to cAMP elevation, such as circadian rhythm, light signaling, ion and nitrate transport, proteasome-mediated ubiquitin-dependent protein catabolic process, mRNA catabolic process, and cell wall biogenesis et al., which suggest the diverse regulatory functions of cAMP signaling in plants. We selected and listed the CRGs of these biological processes in Table 1. Obviously, the BP terms included three distinct groups that may reflect the overall feature of cAMP in functions related to transcription, hormone signaling, and environmental responses (Figure 3A).

### 3.3. Functional Comparison between Up- and Down-Regulated CRGs in Plants

**Figure 4:**
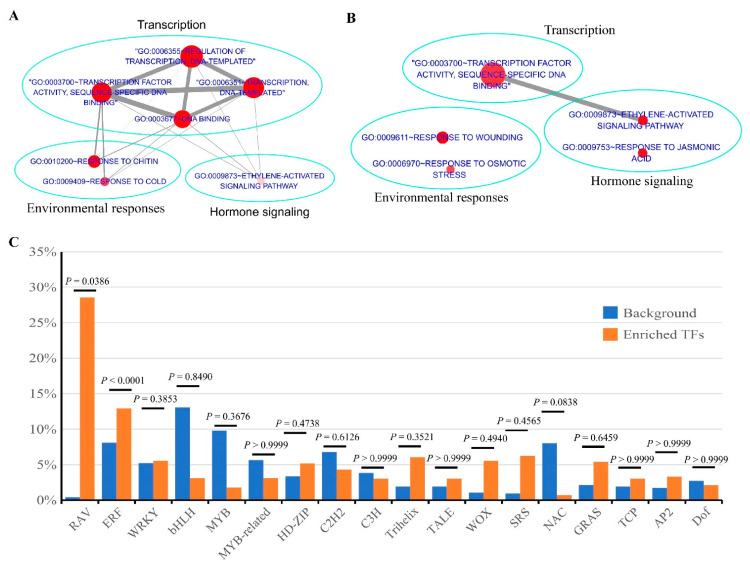
Functional comparison between up- and down-regulated cAMP-responsive genes (CRGs). (**A**) Enrichment map of up-regulated CRGs (Supplementary Data S1). The significantly enriched Gene Ontology (GO) functional terms (Supplementary Data S3) were depicted as a network with the node size, node color, and edge width corresponding to the number of genes assigned to the given term, the false discovery rate (FDR) (smaller value in darker color), and the number of overlapped genes between the two connected terms, respectively. (**B**) Enrichment map of down-regulated CRGs (Supplementary Data S1). See description for (**A**). (**C**) Over-representation analysis of transcription factor (TF) families in the CRGs. Fisher’s exact test was performed to determine *p*-values for the significance of difference.

The up- and down-CRGs were analyzed separately for functional enrichment profile using DAVID, and the resulting significantly enriched terms (Figure 4A,B; Supplementary Data S3) at the threshold of FDR < 0.05 were displayed as a network to identify biological themes using EnrichmentMap [86]. Both the up- and down-CRGs were significantly enriched with functions related to transcription factor activity (GO:0003700) and the ethylene-activated signaling pathway (GO:0009873), suggesting a peculiar association of cAMP elevation with these functions. In addition, the up-CRGs were significantly associated with regulation of transcription (GO:0006355), transcription (GO:0006351), DNA binding (GO:0003677), responses to chitin (GO:0010200), and cold (GO:0009409), whereas the down-CRGs were significantly associated with responses to jasmonic acid (GO:0009753), wounding (GO:0009611), and osmotic stress (GO:0006970). Clusters of highly related GO terms by gene overlap within the enrichment networks clearly indicated that the functions of cAMP elevation were most notably associated with transcription, and relevant to environmental responses and hormone signaling (Figure 4A,B).

Accordingly, we identified 23 up-CRGs and 39 down-CRGs belonging to transcription factors (TFs) using PlantTFDB and other available TFs databases [87,88], which are attributed to 18 TF families. These TFs accounted for 14.5% of the CRGs and were listed in Table 2, along with five CRGs involving transcriptional regulation; in contrast, only 6.3% of the protein-coding genes in the *Arabidopsis* genome (TAIR10) belong to TFs, which were classified into 58 families in the PlantTFDB database (http://planttfdb.cbi.pku.edu.cn/ (accessed on 24 July 2020)). Statistical analyses using Fisher’s exact test indicated that the families of ETHYLENE RESPONSIVE FACTOR (ERF) and RELATED TO ABSCISIC ACID INSENSITIVE 3/VIVIPAROUS 1 (RAV) were significantly overrepresented among the TFs in the CRGs (Figure 4C), which comprised two subfamilies within the AP2/ERFs superfamily [89]. Thus, cAMP signaling seems to preferentially regulate TFs of the ERFs and RAV families.

### 3.4. Identification of Biological Pathways Associated with Cellular cAMP Elevation in Plants

**Figure 5:**
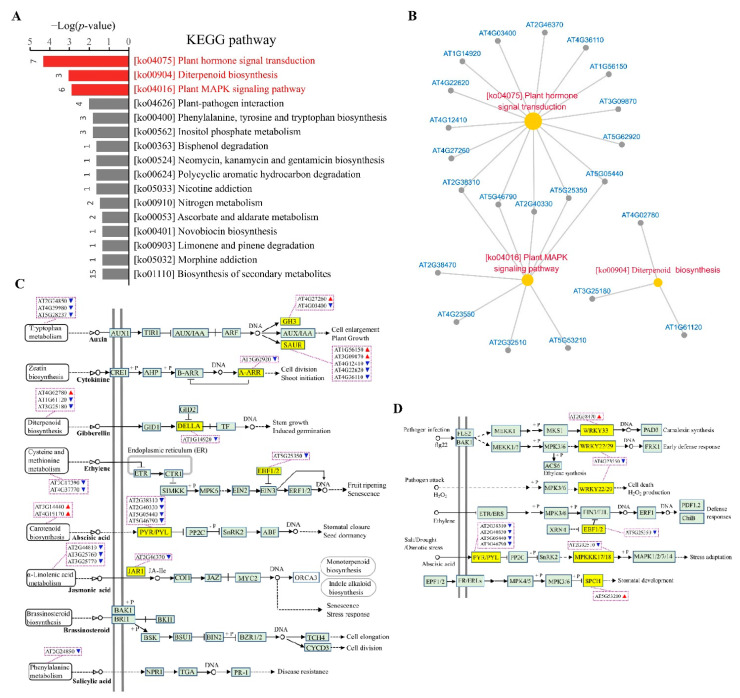
Biological pathways of cAMP-responsive genes (CRGs). (**A**) Bar plot showing enriched KEGG (Kyoto Encyclopedia of Genes and Genomes) pathways of CRGs (Supplementary Data S1 and S4) at the cutoff of hypergeometric test *p* ≤ 0.05. Red bars indicate significant enrichment at the false discovery rate (FDR) < 0.05. Shown on the top of each bar is the count of CRGs with KEGG IDs. (**B**) Network representation of the significantly enriched KEGG pathways (yellow circular nodes) of CRGs (grey circular nodes; gene IDs in blue) showing relationships among them. (**C**) CRGs mapping of plant hormone signal transduction pathway (KEGG: ath04075). (**D**) CRGs mapping of plant MAPK signaling pathway (KEGG: ath04016). In (**C**,**D**), genes highlighted by yellow color in the pathways are pointed out by the associated CRGs in a rectangle box of dotted lines, and the red and blue triangles following the CRGs indicate up- and down-regulation, respectively. Shown in (**C**) are also the CRGs related to the biosynthesis of hormones (left side of double vertical line).

All the CRGs were analyzed using the Kyoto Encyclopedia of Genes and Genomes (KEGG) database (https://www.genome.jp/kegg/ (accessed on 2 Dec 2019)), resulting in the identification of 91 KEGG pathways (Supplementary Data S4). Only three of them were significantly enriched at the level of FDR < 0.05, including plant hormone signal transduction (ko04075), plant MAPK signaling pathway (ko04016), and diterpenoid biosynthesis (ko00904) (Figure 5A), wherein the former two pathways were closely related by sharing five CRGs (Figure 5B). CRGs mapping in the pathways demonstrated an extensive association of cellular cAMP elevation with the signaling cascades of various phytohormones (auxin, cytokinin, gibberellic acid, ethylene, ABA, SA, and JA), biotic and abiotic stress responses, and stomatal development (Figure 5C,D).

While the above three pathways were identified to show significant association with cellular cAMP elevation as determined by the artificial criteria, some of the remaining pathways are known to be an integral part of cAMP signaling network in animals [90]. For example, calcium signaling pathway (ko04020) that was mapped with two CRGs encoding members of phospholipase C (PLC) family, PLC1 (AT5G58670) and PLC7 (AT3G55940); the phosphatidylinositol (PtdIns) signaling system (ko04070) that was involved with the CRGs including PLC1, PLC7, and ITPK3 (AT4G08170); and the inositol phosphate (IP) metabolism (ko00562) that was associated with the CRGs of PLC1, PLC7, and MIOX2 (AT2G19800). Clearly, these data supported the intrinsic interconnection between cAMP and lipid signaling pathways. Some CRGs were mapped in the pathways of phenylpropanoid biosynthesis (ko00940), glutathione metabolism (ko00480), and glycolysis/gluconeogenesis (ko00010), confirming the relevance of these pathways to cAMP signaling as previously reported in plants [21,50,64,91]. It was noted that all these pathways were further revealed to be significant in the following section using Gene Set Enrichment Analysis (GSEA) [74]. Thus, we are confident that this dataset of biological pathways endorsed by the CRGs may provide valuable insights into cAMP signaling in plants, such as the linkages with nitrogen metabolism (ko00910), plant–pathogen interaction (ko04626), ascorbate and aldarate metabolism (ko00053), biosynthesis of secondary metabolites (ko01110), biosynthesis of amino acids (ko01230), endocytosis (ko04144), spliceosome (ko03040), RNA degradation (ko03018), and protein processing in endoplasmic reticulum (ko04141).

It was noted that only 68 CRGs were annotated in the KEGG pathway database, which accounted for only a small part (15.9%) of all the CRGs (Supplementary Data S1). Of the remaining CRGs, CNGC2 (AT5G15410) was down-regulated, supporting the association of cAMP elevation with the relevant pathways of CNGCs, which are well-established effectors of cAMP signaling in animals [67]; βCA3 (AT1G23730) was up-regulated, which functions to catalyze rapid interconversion of CO_2_ and H_2_O into bicarbonate [92,93], supporting the well-established notion in animals that sAC mediates cAMP signaling in response to bicarbonate [17]. In addition, AtKUP7 was among the CRGs. It was previously found that both AtKUP5 and AtKUP7 each contain a functional AC catalytic center, and the AC-mediated cAMP production has been shown to play an essential role in the regulation of AtKUP5 for K^+^ acquisition and translocation [38,39]. Together, all these data conferred the CRGs and thus their functionally related pathways with a high fidelity in association with cAMP signaling.

### 3.5. Identification of cAMP-Responsive Gene Sets in Plants

**Figure 6:**
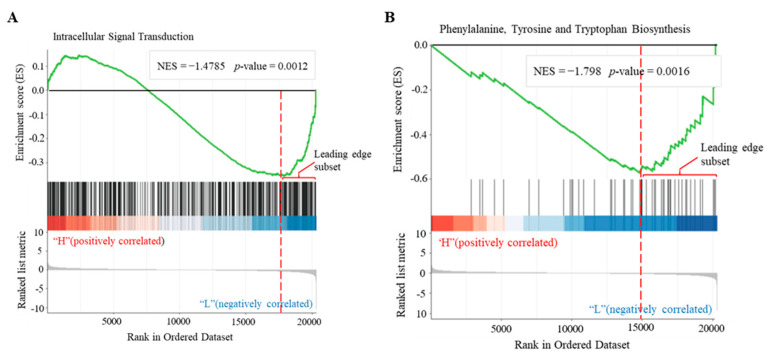
GSEA plots of representative cAMP-responsive gene sets. (**A**) Intracellular signal transduction (GO: 0035556). (**B**) Phenylalanine, tyrosine, and tryptophan biosynthesis (KEGG: ko00400). GSEA: Gene Set Enrichment Analysis; NES: normalized enrichment score; *p*-value: nominal *p*-value of the enrichment score (ES); leading edge subset: the core subset genes that contribute most to the enrichment result. The red dotted line marks the position of maximum enrichment score occurred. GO: Gene Ontology; KEGG: Kyoto Encyclopedia of Genes and Genomes.

Cellular signaling often involves modest or subtle but coordinated changes in the expression of functionally related genes. Thus, we performed GSEA to identify cAMP-responsive gene sets per the statistically significant and concordant differences between the two biological states of LD versus LB. Consequently, a total of 3263 GO-based gene sets (Supplementary Data S5) were obtained, and 161 (4.93%) of them were significant at the threshold of FDR < 0.25 per the recommendation of GSEA developer. In contrast, a total of 231 KEGG-based gene sets (Supplementary Data S5) were obtained, and eight (3.46%) of them were significant. The top-ranking significant gene sets derived from GO terms included intracellular signal transduction (GO:0035556), apoplast (GO:0048046), response to oxidative stress (GO:0006979), glycosyl compound metabolism (GO:1901657), and response to wounding (GO:0009611). The significant gene sets derived from KEGG pathways included phenylalanine, tyrosine and tryptophan biosynthesis (ko00400), α-linolenic acid metabolism (ko00592), drug metabolism (ko00983), PtdIns signaling system (ko04070), IP metabolism (ko00562), estrogen signaling pathway (ko04915), biosynthesis of antibiotics (ko01130), and pyrimidine metabolism (ko00240). These results not only provided complementary data to confirm the biological pathways identified from the CRGs as described above, but also revealed additional biological processes significantly relevant to cAMP signaling, such as brassinosteroid homeostasis (GO:0010268), pattern specification process (GO:0007389), meristem development (GO:0048507), ion homeostasis (GO:0050801), nutrient reservoir activity (GO:0045735), microtubule motor activity (GO:0003777), cell division (GO:0051301), photomorphogenesis (GO:0009640), RNA polymerase activity (GO:0097747), cell–cell signaling involved in cell fate commitment (GO:0045168), and others. In the GSEA, the leading-edge subset within each gene set was determined to represent core enriched genes (Figure 6), and thus we selected and listed those significant GO-based gene sets containing more than 40 core subset genes, along with five significant KEGG-based gene sets in Supplementary Table S2 (for a list of core genes, see Supplementary Data S5), which included a total of 44 gene sets to serve the needed data resources of cAMP-responsive gene sets for use with GSEA-related analyses and other applications in future.

### 3.6. Co-Expression Networks and Hub Genes Associated with Cellular cAMP Elevation in Plants

**Figure 7:**
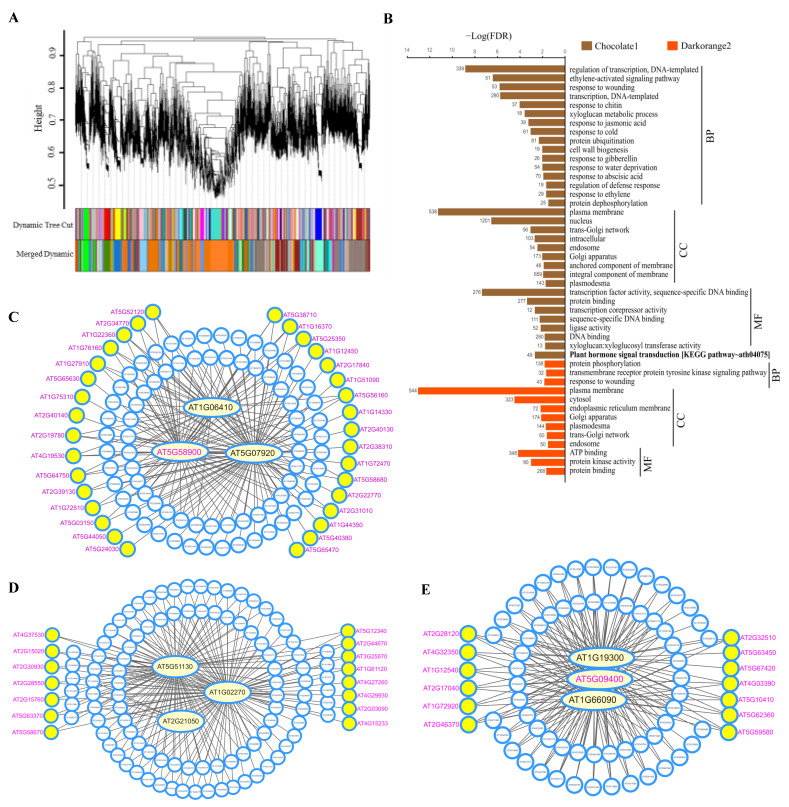
Co-expression networks and hub genes associated with endogenous cAMP elevation in plants. (**A**) Hierarchical clustering tree of RNA-seq expression data set (as shown in Figure 2B) generated by WGCNA [75]. Each leaf in the tree represents one gene, and the major tree branches constitute 35 modules (Supplementary Table S3) labeled by the colored panel beneath the dendrogram. (**B**) Bar plot showing the significantly enriched GO (Gene Ontology) terms and KEGG (Kyoto Encyclopedia of Genes and Genomes) pathways (Supplementary Data S6) in the co-expression modules of “chocolate1” (indicated by brown bars) and “Darkorange2” (red bars) at the threshold of false discovery rate (FDR) < 0.05. The KEGG pathway is marked in bold. BP: biological processes; MF: molecular functions; CC: cellular components. (**C**) Network representation of the co-expression module “chocolate1”. (**D**) Network representation of the co-expression module “darkorange2”. (**E**) Network representation of the co-expression module “cornflowerblue”. In (**C**) to (**E**), for an easy visualization, only the top 10% of genes with highest K_ME_ in each module (Supplementary Data S7) were used to construct the networks using Cytoscape [76]. Hub genes are determined with at least four depicted connections in the network and positioned in the central part by oval nodes, and other genes are denoted by circular nodes. Gene IDs in pink indicate the cAMP-responsive gens (CRGs).

To better understand cAMP signaling at a network level, the RNA-seq data were subjected to WGCNA [75]. All the detected genes by RNA-seq were used to build the correlation matrix by calculating the pairwise Pearson correlations of gene expression across all samples. A total of 35 distinct modules (Figure 7A; Supplementary Table S3) were determined with average linkage hierarchical clustering by the dynamic tree-cutting algorithm, and six of them showed a significant module–trait relationship (*R* = −0.88 or 0.88, *p* = 0.02). However, we found that the CRGs were mainly distributed within the three significant modules of “chocolate1”, “cornflowerblue”, and “darkorange2”, and all other modules contained few CRGs (Supplementary Table S3). “chocolate1”, “cornflowerblue”, and “darkorange2” contained 284, 31, and 88 CRGs, respectively, which accounted for a total of 94.4% of the CRGs, supporting their inherent associations with cellular cAMP elevation.

Interestingly, “chocolate1” and “darkorange2” comprised 3119 and 3183 genes, respectively, representing the two largest ones among the 35 modules that averaged out 808 gene per module; in contrast, “cornflowerblue” comprised 786 genes. These three modules contained 7088 genes in total, which accounted for 25.1% of all the detected genes in this study, implying that cellular cAMP elevation was associated with extensively coordinated gene expression and involved in the modest or subtle expression changes of a great number of genes beyond the CRGs. We performed functional enrichment analyses with these modules using DAVID [85]. Consequently, the enrichment profile of “chocolate1” was highly comparable to the CRGs, showing the most significantly enriched GO terms related to regulation of transcription and transcription factor activity, and plant hormone signal transduction being the only significantly enriched KEGG pathway; in contrast, “darkorange2” showed significant enrichment of GO terms related to protein phosphorylation and ATP binding, but no significant enrichment of KEGG pathways (Figure 7B; Supplementary Data S6). However, “cornflowerblue” displayed no significantly enriched functions (Supplementary Data S6). These results seemed to suggest the distinct features of “chocolate1” mainly related to transcriptional regulation and “darkorange2” associated with phosphorylation, whereas “cornflowerblue” likely represented a theme of physiological responses per the functions of CRGs and hub genes within the module as identified below.

Co-expression networks were constructed with each node standing for a gene and the edges between genes representing co-expression correlations indicated by the K_ME_ (eigengene connectivity) value and visualized using Cytoscape [76]. For an easy visualization, the top 10% of genes with highest K_ME_ (Supplementary Data S7) were used to depict a subnetwork, wherein those genes with at least four depicted connections in the network were determined as the hubs and positioned in the central part (Figure 7C–E; Supplementary Table S4). Among the hubs of “chocolate1” were DIV1 (AT5G58900), which belongs to the CRGs and is a MYB transcription factor responding to various phytohormones, elicitor and stress [94]; TPS7 (AT1G06410), which plays regulatory functions associated with trehalose metabolism in the integration of different metabolic, hormonal, and developmental signals [95,96]; and DGK1 (AT5G07920), which is a pivotal enzyme for phospholipid signaling [97]. The hub genes of “darkorange2” included LAX2 (AT2G21050), which is a member of the AUX1 LAX family of auxin influx carriers and plays critical roles in patterning or morphogenesis [98], the calcium-binding endonuclease/exonuclease/phosphatase family protein (AT1G02270) [99], and the S-adenosyl-L-methionine (SAM)-dependent methyltransferase (AT5G51130) that utilizes the methyl donor SAM as a cofactor to methylate various biomolecules and engages an important role of adenosine kinase [100]. The hub genes of “cornflowerblue” comprised of the NBS-LRR protein (AT1G66090) implicated in defense responses [101]; PARVUS (AT1G19300), which acts as the glycosyltransferase required for xylan biosynthesis [102]; and AtKUP7, which is among the CRGs and plays crucial roles in K^+^ uptake and translocation under limited K^+^ conditions [103].

### 3.7. Phenotypic Effects of Cellular cAMP Elevation in Plants

**Figure 8:**
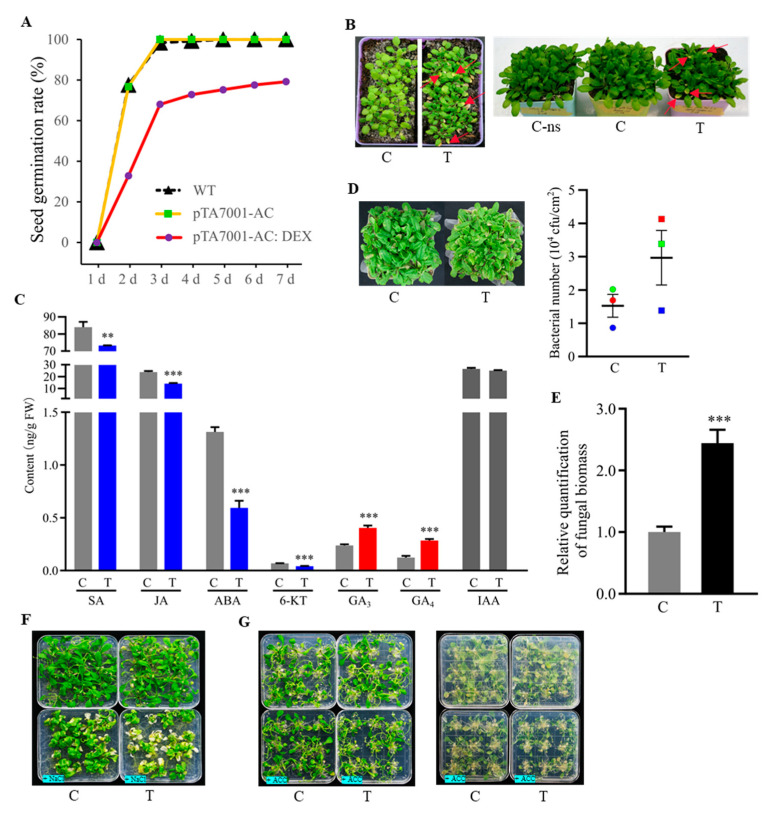
Phenotypic effects of cellular cAMP elevation in plants. (**A**) Inhibition of seed germination. Seeds obtained from *AC* transgenic plants were germinated with dexamethasone treatment (pTA7001-AC: DEX) or no treatment (pTA7001-AC), compared to wild type (WT) control. (**B**) Pre-mature leaf senescence. Left panel: 12-day-old *AC* transgenic seedlings were thoroughly sprayed with DEX (T) or mock control (C) and photographed 10 days later. Right panel: 16-day-old seedlings were treated as described in the left panel, except the spray repeated once in the other day and a non-spraying control (C-ns) and photographed 19 days later. Arrows point to representative symptoms of yellowing at the leaf tips. (**C**) Altered levels of phytohormones. The contents of phytohormones were measured in 18-day-old *AC* transgenic seedlings at 0 h (C) and 24 h (T) after spraying DEX. Data are Ave ± SD (*n* = 3), two-tailed Student’s *t*-test ** *p* < 0.01, and *** *p* < 0.001. (**D**) Compromised resistance to bacterial infection. Four- to five-week-old *AC* transgenic plants were inoculated with *Pst* DC3000. Left panel: a representative photograph taken five days after inoculation, showing more severe infection symptoms with DEX treatment (T) versus mock control (C). Right panel: determination of bacterial growth in leaves 10 days after inoculation. Data are Ave ± SE from three separate experiments indicated by different colors in the figure. (**E**) Compromised resistance to fungal infection. Detached leaves from 34-day-old *AC* transgenic plants were inoculated with *V. dahliae* strain Vd991 under conditions of DEX treatment (T) or mock control (C), and the relative fungal biomass was determined 10 days after inoculation. Data are Ave ± SD (*n* = 3), two-tailed Student’s *t*-test *** *p* < 0.001. (**F**) Compromised resistance to salt stress. 12-day-old *AC* transgenic seedlings were transplanted to growth medium containing DEX (T) or mock control (C) with the addition of 100 mM NaCl (bottom panels) or not (upper panels), photograph taken 20 days later. (**G**) Increased sensitivity to ethylene. As performed in (F), except the treatment of 100 µM ACC (1-aminocyclopropane-1-carboxylate), photographs of the shoots (left panels) and roots (right panels) were taken 20 days later.

The *AC* transgenic plants did not show any visible difference from WT plants under a resting state (no DEX treatment). However, germination rates of seeds from *AC* transgenic plants (pTA7001-AC: DEX) were only 68.0% and 79.2% on the third and seventh day after germination under the condition of DEX treatment, respectively, compared with almost 100% germination on the third day under the condition of without DEX treatment for seeds of both WT and pTA7001-AC transgenic plants (Figure 8A), indicating the inhibitory effect of cAMP elevation during seed germination. When young *AC* transgenic seedlings were thoroughly sprayed once with DEX, chlorotic symptoms on the leaf tips, a typical feature of pre-mature leaf senescence, were observed 10 days later after the spray; however, it required multiple spraying on intervals to see the symptoms on older seedlings (Figure 8B), suggesting a regulatory role of cAMP related to the process of senescence. We measured and compared the levels of phytohormones between the whole plant tissue samples of 18-day-old *AC* transgenic seedlings taken at 0 h (C) and 24 h (T) after spraying DEX, indicating that induction of cAMP elevation reduced SA, JA, ABA, and 6-KT, increased GA_3_ and GA_4_, but did not alter IAA (Figure 8C).

The *AC* transgenic plants were also characterized under growth conditions of biotic and abiotic stresses, as well as hormonal treatments. Through comparison of *AC* transgenic plants under conditions of DEX treatment (T) and mock control (C), we found that induction of cAMP elevation increased bacterial growth as well as fungal growth in leaves (Figure 8D,E). Additionally, the induction of cAMP elevation caused an enhanced susceptibility of *AC* transgenic seedlings to salt stress (Figure 8F) and an inhibitory growth effect to the exposure of ethylene precursor 1-aminocyclopropane-1-carboxylate (ACC) (Figure 8G). In contrast, we did not observe the apparent effects of cAMP elevation with all other tested conditions, including methyl jasmonate (MeJA), SA, GA_3_, α-naphthyacetic acid (NAA), 6-benzylaminopurine (BAP), and mannitol (Supplementary Figure S2). These results suggested remarkable roles of cAMP in plant defense, salt stress, and ethylene responses. However, induction of cAMP elevation caused detrimental effects, which generally became visible after a relatively long period of DEX treatment, especially for the exposure to salt stress and ethylene, implying a comprehensive regulatory role of cAMP in plant development.

## 4. Discussion

In the present study, our results contributed an important leap toward the understanding of cAMP signaling in plants and provided highly valuable data resources in urgent need for the community to explore. We found that the molecular mechanism of cAMP signaling in plants was operative in some way analogous to that in animals and other living organisms. To support our conclusions, we have also performed RNA-seq analyses by creating transgenic *Brassica napus* plants using the same AC construct in this study, and comparable results were obtained (manuscript in preparation).

### 4.1. Interaction of cAMP and Hormones in Plants

cAMP was initially discovered by studying hormone-mediated glucose metabolism in mammals, and its intracellular elevation by activation of ACs modulates many cellular processes and causes physiological responses including the production of certain other hormones such as steroids [1]. In plants, interactive effects between cAMP and phytohormones were previously documented; however, little is known about the underlying molecular mechanisms until now. Exogenous cAMP, in combination with auxin, exhibited the cell-division-promoting activity of cytokinin in plan tissue culture [46,104]; it also mimicked the action of IAA in activating the de novo synthesis of tryptophan oxygenase, while IAA seemed to stimulate cAMP synthesis [44,105]. In combination with a low concentration of GA_3_, exogenous cAMP promoted seed germination [43], whereas GA_3_ seemed to modulate cAMP level during seed germination [42]. ABA inhibited cAMP-induced seed germination [43], whereas cAMP completely reversed exogenous ABA-induced inhibition of stomatal opening [106]. Recently, both cAMP and forskolin (a specific activator of ACs) were shown to significantly stimulate SA level, whereas the inhibitor (2′,5′-dideoxyadenosine) of ACs dramatically reduced toxin-induced SA increase in *Arabidopsis* [68]. In addition, JA induced the rapid elevation of cellular cAMP in plants [69].

In line with these previous findings, here we showed that cellular cAMP elevation modulated the levels of various phytohormones in plants (Figure 8C). Moreover, the CRGs were significantly enriched in functions related to plant hormone signal transduction pathway and responses to various phytohormones (Figure 3A and Figure 5A,C). Some CRGs responsible for the biosynthesis or homeostasis of phytohormones include PIN6 (AT1G77110) and PID (AT2G34650) for auxin [107]; JAR1 (AT2G46370), AOC1 (AT3G25760), and AOC2 (AT3G25770) for JAs [108]; GA1 (AT4G02780) for GA [109]; ACS8 (AT4G37770) for ethylene [110]; and LOG7 (AT5G06300) for bioactive cytokinin production [111]. These findings provided direct evidence to support an extensive interaction of cAMP and phytohormones in plants, and identification of these CRGs may allow us to revisit previous studies for elucidating the underlying molecular mechanisms. Recent advances increasingly highlighted the formation of a complex phytohormone crosstalk network, which involves small molecules and TFs [112,113]. Consistently, numerous TFs or transcriptional regulators among the CRGs act pleiotropically in the network, such as GAI mediating crosstalk of GA, ethylene and JA signaling [112], and that ZFP8 (AT2G41940) integrating cytokinin and GA signaling in the regulation of epidermal cell fate [114]. Taken together, cAMP may be a key player in the formation and coordination of phytohormone network in plants.

### 4.2. Regulatory Roles of cAMP Signaling during Gene Expression in Plants

Transcriptional regulation is a hallmark of cAMP signaling across different kingdoms of life [5,20,115], but evidence was rarely documented in plants until now. It was reported that exogenous cAMP stimulated in vitro RNA and protein synthesis in chickpea seedlings [116] and induced rapid expression of several genes in the phenylpropanoid pathway in *Arabidopsis* [91]. Here, we showed that the CRGs were most significantly enriched in functions related to transcription, and 14.5% of them are TFs (Table 2). Intriguingly, the TFs were overrepresented with ERF and RAV family members (Figure 4C), which belong to the AP2/ERFs superfamily that acts in the signaling events of many phytohormones and various stress responses [89]; moreover, almost one third of the TFs are ERF family members, which are specific to plants and differentially regulated by ethylene [117]. These results are well in line with the effects of cAMP elevation on the levels of phytohormones (Figure 8C) and their notable interaction with ethylene on the growth phenotypes (Figure 8G). While it is well known that the major pathway of cAMP signaling is to activate TFs through PKA phosphorylation in animals [118], our results highlighted a similar mechanism operative in plants. Among TFs in the CEGs, RAP2.6 (AT1G43160) responds to stress hormones (JA, SA, ABA, and ethylene) in addition to salt and drought [119]; WRKY33 (AT2G38470) is a key transcriptional regulator of hormonal and metabolic responses toward pathogen infection [120]; JACKDAW (AT5G03150) regulates tissue boundaries and asymmetric cell division [121]; SPEECHLESS (AT5G53210) is required for the stomatal initiation and development [122]; SHI (AT5G66350) acts in gynoecium development [123]; BBX32 (AT3G21150) modulates light signaling [124]; CDF5 (AT1G69570) contributes to photoperiodic flowering by modulating an underlying diurnal rhythm in CONSTANS transcript levels [125]; HAT4 (AT4G16780) acts as a developmental regulator to control phenotypic changes [126]; ZFP5 (AT1G10480) modulates root hair initiation and elongation in response to cytokinin and ethylene signals [127], whereas ZFP8 regulates trichome initiation via integration of cytokinin and GA signaling [114]; and CRF3 (AT5G53290) functions in the development of embryos, cotyledons, and leaves in response to cytokinin [128]. In addition, SMXL8 (AT2G40130) acts as a transcriptional regulator in the branching control in response to strigolactone [129]. While the remaining TFs or transcriptional regulators (Table 2) remain poorly characterized in functions, it is evident that cAMP signaling targets transcriptional regulation of TFs that are closely associated with the phytohormone signaling network and plays diverse functions in plant development and environmental responses.

The degradation of mRNA is a vital aspect of gene expression [130]. Some CRGs are functionally related to mRNA decay, including SOV (AT1G77680) acting selectively in the decay of decapping substrates [131], CAF1-5 (AT1G61470) involving shortening of the poly(A) tail and responding transiently to hormones and stresses [132], TSN2 (AT5G61780) playing a role in stress-induced mRNA decapping [133], and BRN2 (AT1G03457) functioning in 3′ UTR-dependent decay of SOC1 mRNA [134]. Thus, cAMP signaling may play regulatory roles via the mechanism of mRNA decay for fine-tuned cellular responses by enabling rapid changes of mRNA populations in plants. The regulation of protein stability is critical in plant responses to environmental signals, which is largely mediated by the ubiquitin-proteasome system (UPS), especially the functions of E3 ligases as regulators of phytohormone signaling pathways for a coordinated response [135]. The CRGs included aspartyl protease (AT4G16563) and a set of U-box or F-box type E3 ubiquitin ligases, comprising PUB29 (AT3G18710), PUB45 (AT1G27910), RING/U-box superfamily protein (AT5G47610), SGR9 (AT5G02750), SNIPER1 (AT1G14200), EBF2 (AT5G25350), PP2-A14 (AT5G52120), KMD2 (AT1G15670), KMD4 (AT3G59940), F-box/kelch-repeat protein (AT1G14330), HUP6 (AT3G27220), and BTB/POZ domain-containing protein (AT2G30600), most of which have been documented to play diverse roles in plant development, stress responses, phytohormone signaling, and secondary metabolites [136,137,138,139,140,141]. Recently, it was reported that cAMP deficiency in tobacco BY-2 cells caused differential expression of many proteasome subunits [50]. These results together support the notion that cAMP signaling is involved in the regulation of protein stability in plants. Consistently, a recent study showed that a chaperone-assisted ubiquitin system mediated the feedback control of cAMP effector signaling in animals [142].

It was noted that among the CRGs were CDKG1 (AT5G63370), which is associated with the spliceosome and regulates pre-mRNA splicing of *CALLOSE SYNTHASE5* [143], and LOS1 (AT1G56070), which is essential for protein synthesis [144], implying the linkage of cAMP signaling with pre-mRNA processing and translation in plants. In line with these results, it was shown that cAMP deficiency in tobacco cells resulted in differential expression of translation initiation factors [50], and that the translation initiation factor 4A1 (EIF4A1; AT3G13920) was among the cAMP binding proteins [70], as well as that numerous ribosomal proteins were differentially expressed by cAMP treatments in plants [50,63,64]. Accordingly, differential expression proteins have recently been identified under various regimes of cAMP treatment in plants [21,50,63,64].

### 4.3. The Pathways and Interplay Network of cAMP Signaling in Plants

The CRGs were significantly enriched with three KEGG pathways (Figure 5A). Of them, the plant hormone signal transduction pathway was also significantly enriched with the “chocolate1” module derived from WGCNA (Figure 7B), together with the finding that cAMP elevation altered the levels of phytohormones (Figure 8C), strongly supporting an innate connection of cAMP and hormonal signaling. While hormonal pathways are interconnected by a network of interactions and feedback circuits that determines the final outcome of the individual hormone actions in plants [145], we suggest that cAMP signaling may act through hijacking or modulating hormone signaling network in plants. The plant MAPK signaling pathway plays key roles in the transduction of environmental and developmental signals through phosphorylation of downstream signaling targets [146], which was significantly enriched with the CRGs and showed an interplay with plant hormone signal transduction pathway by sharing some CRGs (Figure 5A,B). Most of the CRGs in the MAPK signaling cascades are TFs and act downstream of protein kinases (Figure 5D). It has been demonstrated that cAMP activated TFs by phosphorylation through both PKA and a time delayed MAPK pathway in animals [118], whereas the interaction between cAMP and MAPK signaling cascades in the regulation of fungal development and virulence was also uncovered [147]. Interestingly, diterpenoid biosynthesis was among the significantly enriched pathways (Figure 5A), which was mapped with three up-regulated CRGs, including CPS (AT4G02780) acting as the initial cyclase in GA biosynthesis [148], AtGES (AT1G61120) and CYP82G1 (AT3G25180) both functioning for the key enzymes to produce acyclic homoterpenes TMTT and DMNT [149]. These results indicated the relevance of cAMP signaling with the biosynthesis of phytohormones and secondary metabolites. In consistency, the results of GSEA revealed the significant enrichment of another two closely related pathways (Supplementary Table S2). Of them, the pathway of phenylalanine, tyrosine, and tryptophan biosynthesis is implicated in the production of a variety of secondary metabolites, including plant pigments (e.g., flavonoids and proanthocyanidins), defense compounds (lignins and cutin), and hormones (auxins) [150], which was mapped with three CRGs, i.e., TAT3 (AT2G24850), DHS1 (AT4G39980), and tryptophan synthase (AT5G28237); in contrast, the pathway of α-linolenic acid (ALA) metabolism is related to the biosynthesis of JAs, which modulate the production of secondary metabolites [151,152], and it was associated with the CRGs including DND1 (AT2G44810) that catalyze the initial step of JA biosynthesis [153], AOC1 and AOC2 both being the allene oxide cyclase [154]. Obviously, these results were in line with previous findings that both cAMP and forskolin stimulated the biosynthesis of phytoalexins (low molecular weight antimicrobial compounds including diterpenoids), which is controlled by transcriptional regulation, phytohormones, and multiple signal transduction pathways [53,54,155], and that cAMP regulated the phenylpropanoid pathway in *Arabidopsis* [91].

Crosstalk between cAMP and lipid signaling is well established in animals [90]. For example, diacylglycerol (DAG) stimulates PKC_δ_ that in turn activates AC and cAMP production [156]. Both PtdIns signaling system and IP metabolism were among the significant pathways identified by GSEA (Supplementary Table S2). The former pathway involves sequential phosphorylation of PtdIns by lipid kinases, leading to the production of phosphoinositides, while the activation of PLC by environmental stimuli catalyzes the hydrolysis of phosphatidylinositol-(4,5)-bisphosphate (PIP2) into inositol 1,4,5-trisphosphate (IP3) and DAG [157,158]. IP metabolism is intricately tied to the PtdIns signaling system, as at least one portion of the IP signaling pool is derived from hydrolysis of PIP2 by PLC [159]. Among the CRGs involving these pathways, MIOX2 (AT2G19800) is a *myo*-inositol oxygenase and plays essential roles in energy/nutrient sensing [160]; ITPK3 (AT4G08170) is an inositol 1,3,4-trisphosphate 5/6-kinase that phosphorylates various IPs [161], whereas both PLC1 and PLC7 are the phosphoinositide-specific PLC family members that catalyze the hydrolysis of PIP2 to IP3 and DAG [162]. Additionally, SFH14 (AT5G56160) is among the PtdIns transfer proteins (PITPs) that play critical roles in integrating diverse territories of the lipid metabolome with phosphoinositide signaling in plants [163]. Over recent years, it has become increasingly evident that the PtdIns signaling system plays critical roles in mediating the effects of various hormones and cell responses to environmental stimuli in plants [158,164].

Estrogens are a group of steroid hormones that regulate target gene transcription in the signaling pathways and generate the linkages with cAMP, DAG, and IP3 signaling in animals [165]. The estrogen signaling pathway was identified with significant enrichment (Supplementary Table S2), which harbored the CRG being an important chaperone protein HSC70.3 (AT3G09440) that plays a role in defense responses [166]. In plants, brassinosteroids (BRs) are the known steroid hormones, which regulate the transcription of target genes via the BES1/BZR1 family transcription factors [167]. Very interestingly, the BR receptor BRI1 contains a guanylate cyclase catalytic center and generates cGMP to modulate its own signaling activity [168], while BRI1-associated receptor kinase 1 (BAK1) directly interacts with and phosphorylates CNGC20 [169]. Crosstalk between cAMP and cGMP signaling is common in animals [170]. Some CRGs are associated with BR metabolism or homeostasis, including SMT3 (AT1G76090), which is a sterol methyltransferase in the phytosterol biosynthetic pathway [171], CYP702A3 (AT4G15310) being a member of cytochrome P450s family [172], and a homolog of DRL1 (AT2G40230) [173].

### 4.4. The Association of Ion Channels and Transporters with cAMP Signaling in Plants

Accumulated evidence supported the notion that the regulation of CNGCs by direct binding of cAMP and Ca^2+^/calmodulin may be conserved between animals and plants [55,174,175]. Intriguingly, AtCNGC2 (AT5G15410) was among the down-CRGs (Supplementary Data S1). Both JA and pathogen infection stimulated cAMP elevation that in turn activated AtCNGC2, resulting in cytosolic Ca^2+^ rise and immune responses in *Arabidopsis* [61,69]. Thus, we envision that the stimulated elevation of cAMP activates AtCNGC2 to mediate Ca^2+^ influx, but also triggers a feedback mechanism to control this activity by down-regulated expression of *AtCNGC2*, which may ultimately contribute to the generation of calcium signatures that are commonly observed in plant responses to environmental and endogenous cues [176]. While calcium signatures reflect the coordinated action of various Ca^2+^ influx and efflux systems including channels, pumps, and exchangers located at the cellular membranes, the related CRGs are CAX7 (AT5G17860) and CSC1-like proteins (AT1G10090; AT1G62320) that function as calcium-permeable cation channels [177,178]. Together, these results suggest that cAMP targets Ca^2+^ flux and plays a role in the modulation of Ca^2+^ signaling in plants.

Nitrogen (N), phosphorus (P), and potassium (K) are the three main nutrients needed by plants for growth. Plants are capable of perceiving external K^+^ changes and generating signals for the control of K^+^ homeostasis, particularly via the modulation of K^+^ channels and transporters [179]. Recently, both AtKUP5 and AtKUP7 were found to contain a functional AC catalytic center [38,39]. The AC activity of AtKUP5 has been shown to be essential for K^+^ uptake, which causes the flux-dependent cAMP increases, likely signaling for the modulation of K^+^ homeostasis [38]. Intriguingly, AtKUP7 was among the up-CRGs and acted as a hub gene of the “cornflowerblue” module (Figure 7E). Therefore, cAMP signaling may be a key player in the regulation of K^+^ homeostasis in plants. Nitrate serves a major source for N nutrient, but also acts as an important regulatory molecule to form signaling networks in plants [180]. Notably, several nitrate transporters belong to the CRGs, including NPF5.12 (AT1G72140), which mediates nitrate uptake in a pH-dependent manner and acts as vacuolar nitrate efflux transporters [181], NPF7.2/NRT1.8 (AT4G21680) functioning to remove nitrate from xylem vessels [182], NRT2.6 (AT3G45060) playing in the responses to challenges of microorganisms including plant growth-promoting rhizobacteria [183], and NPF2.7/NAXT1 (AT3G45650) acting as nitrate efflux transporter in roots [184]. Thus, cAMP is anticipated to regulate plant nitrate signaling. Taken together, we suggest an important role of cAMP signaling in the regulation of nutrient availability in plants, as is increasingly evident in yeast and animals [185,186].

Sugars are the primary products of photosynthesis in plants, which provide the main carbon and energy source for cellular metabolism and act as hormone-like signaling molecules [187]. Among the CRGs, SWEET16 (AT3G16690) acts as a vacuolar sugar carrier that is tightly regulated to allow optimal development under non-favorable conditions [188]); HKL1 (AT1G50460) interacts with the glucose sensor HXK1 and mediates glucose and ethylene responses [189]; and PMT6/PLT6 (AT4G36670) was suggested to mediate long distance transport of glucose import [190]. In addition, we identified TPS7 (AT1G06410) being a hub gene of “chocolate1” module (Figure 7C), which is the enzyme to synthesize trehalose-6-P (T6P) [191]. T6P is an essential signal metabolite and negative feedback regulator of sucrose levels, forming part of a mechanism to maintain sucrose levels within an optimal range and being functionally comparable to the insulin–glucagon system for regulating blood glucose levels in animals [192]. Plant sugar signaling interfaces with phytohormone signaling network and exhibits interplay with nitrogen in the fine-tuning of plant growth, development, and survival [189,193]. While the interplays of cAMP, hormones, and glucose are well established in animals [194], our results clearly support that these mechanisms are also operative in plants.

In animals, sAC is uniquely regulated by HCO_3_^−^ and Ca^2+^, suggesting it may also contribute to other processes responsive to CO_2_ and/or functions as a metabolic sensor in cells [17]. In plants, βCA1 (AT3G01500) interconverts soluble HCO_3_^−^ to gaseous CO_2_ in chloroplast, controls the supply of CO_2_ to Rubisco, and regulates stomatal closure through HCO_3_^−^ effects on anion channels [93], while HCO_3_^−^ also induces redox tuning in photosystem II for regulation and protection [195]. βCA1 was recently identified as a cAMP binding protein [70]. In the present study, we identified βCA3 among the up-CRGs, which has been shown to play catalytic function in the cytosol [92,93]; in addition, the S-type anion channel SLAH3 (AT5G24030) among the down-CRGs is a key mediator of stomatal closure for the control of CO_2_ sensitivity, which is very sensitive to nitrate, calcium, and ABA in the guard cells [196,197]. A role of cAMP in ABA- and Ca^2+^-mediated signal transduction of stomatal regulation has been previously reported in plants [106]. Altogether, these results support that cAMP signaling may be fundamental to the control of CO_2_ sensitivity through stomatal movement in the epidermis and contributes to the regulation of photosynthesis in plants, which may represent an analogous mechanism of cAMP signaling through the regulation of HCO_3_^−^ between plants and animals.

### 4.5. cAMP-Responsive Gene Sets and Co-Expression Modules in Plants

cAMP mainly plays regulatory functions, which may confer distinct features of coordinated gene expression. In a recent report, a total of twelve cyclic nucleotide binding proteins were identified in *Arabidopsis*, and ten of them were transcriptionally co-expressed [70]. Using WGCNA, we identified three distinct co-expression modules, i.e., “chocolate1”, “darkorange2”, and “cornflowerblue”, which were closely associated with cAMP elevation (Figure 7C–E; Supplementary Table S3). The “chocolate1” module was featured in function particularly related to transcription (Figure 7B), highlighting the important significance of transcription-related activities for cAMP signaling in plants. Protein phosphorylation through kinases is a hallmark of cellular signal transduction, and ATP is required during the process [198]. In animals, a typical feature of cAMP signaling is to target kinases that activate a family of transcription factors for transcriptional regulation [19]. Interestingly, it happened that the module “darkorange2” was featured with enriched function related to phosphorylation (Figure 7B), suggesting that the mechanism of phosphorylation for cAMP signaling is operative in plants. However, the targets of protein kinases for cAMP have not been identified in plants until now. While the module “cornflowerblue” did not show any significantly enriched function, it contained co-expression genes of diverse functions (Figure 7E; Supplementary Table S4), implying a feature likely relevant to physiological responses. Thus, the above three modules seem to represent distinctive stages featuring highly coordinated gene expression during cAMP signal transduction pathway. It was noted that “chocolate1” and “darkorange2” were the largest modules, containing 3119 and 3183 genes, respectively, compared to only 808 genes for an average of all the 35 modules (Supplementary Table S3), highlighting the importance of coordinated gene expression with cAMP signaling. Therefore, it is of significance to characterize the regulatory roles of cAMP signaling from the aspect of functionally related genes that may involve modest or subtle expression changes. To this end, we determined cAMP-responsive gene sets using GSEA (Supplementary Table S2), which provided fundamental data resources for a variety of applications in future.

### 4.6. Phenotypic Effects of cAMP Disturbance in Plants

Phenotypic changes were not observed in transgenic *Arabidopsis* plants that constitutively overexpressed either the *AC* transgene under the control of CaMV35S promoter for increasing cAMP (data not shown in this study) or the cAS for decreasing cAMP availability [21], implying that the regulatory roles of cAMP may involve rigorous feedback control in plants. In a similar study for cGMP signaling, constitutive overexpression of the rat soluble guanylate cyclase (GC) in *Arabidopsis* did not cause any phenotypic difference from WT plants [83]. However, induced overexpression of the *AC* transgene caused premature leaf senescence (Figure 8B). In animals, it has been documented that cAMP accumulation was correlated with senescence [199], and that elevation of cAMP may ultimately contribute to retinal cell death [200]. Leaf senescence is a highly regulated process involving transcription regulators, receptors, and signaling components for hormones and stress responses, as well as regulators of metabolism [201]. Thus, cAMP signaling is obviously linked with the process of leaf senescence, which may suggest its essentiality in plants. Among the CRGs, WRKY53 (AT4G23810) is a central hub in the WRKY network regulating early senescence [202] and AtS40-1 (AT1G29640) acts as a senescence regulator [203], whereas ERD7 (AT2G17840) is a senescence/dehydration-associated protein-like protein and functions in the protection of cellular components [204]. It was previously shown that seed germination was associated with a transient elevation of cAMP, and exogenous cAMP application impacted seed germination by the interactive effects with ABA and GA_3_ [42,43,205]. Here, we found that induced overexpression of *AC* transgene significantly inhibited seed germination (Figure 8A), which may be attributed to the disturbance of native cAMP signaling.

Both biotic and abiotic stresses were found to stimulate transient elevation of cAMP in plants [21,59,61], suggesting the relevance of cAMP signaling with stress responses. However, induction of cAMP elevation enhanced susceptibility to pathogen challenges in *AC* transgenic plants (Figure 8D,E), whereas cAMP deficiency in “cAS” transgenic plants resulted in compromised immune responses [21]. Similarly, it was reported that constitutively elevated cGMP level compromised systemic acquired resistance (SAR) in GC transgenic plants [83]. These results seemed to suggest the essentiality of a tightly controlled cAMP levels in plant growth and development, and thus detrimental effects were observed due to transgenic disruption of native cAMP status in plants. Consistently, the induction of cAMP elevation was also found to compromise responses to salt stress and ACC treatment in *AC* transgenic plants (Figure 8F,G). It was noted that there were more differentially expressed genes of down-regulation than up-regulation in plants under the regime of exogenous cAMP application [64], or endogenous cAMP deficiency [21,50], or induction of cAMP elevation in the present study. Altogether, these results suggest the notion that the precise control of cellular cAMP levels is fundamental to plant growth and development, particularly pertaining to seed germination, senescence, defense, salt stress, and ethylene response. In animals, it has been well shown that cellular cAMP levels are precisely regulated [206], and increases in intracellular cAMP generally suppress innate immune functions [207,208].

### 4.7. A Role of cAMP Signaling in Plant Plasticity

In animals and yeast, cellular cAMP levels display dynamic changes [206,209], as well as rigorous feedback control [210,211]. In fact, cAMP triggers changes in its own concentration [20]. Fluctuation of cAMP levels during growth and development were observed in algae and plants [51,212,213,214,215]. Thus, cellular cAMP levels may have profound effects for the proper functionality of plants, which is evident by the results discussed above. Many biologic processes in plants employ feedback-loop regulation to maintain homeostasis or dynamic equilibrium, including hormone metabolism, morphogenesis, and photosynthesis [195,216,217,218], which obviously warrant an optimal outcome of functions. In animals, it was shown that initially higher levels of cAMP promote cell survival and slow down retinal degeneration, but elevated cAMP levels may ultimately become toxic [200]; additionally, cAMP elevation was found to improve the signal-to-noise ratio in amphibian rod photoreceptors during the sensory transduction of the visual system [219]. Therefore, stimuli-induced transient elevation of cAMP in plants may play beneficial roles for priming an optimized response, likely by triggering the mechanisms of feedback control.

Both sAC and mAC activities are present in plants [23]. Current advances in animals support that cAMP signaling is compartmentalized into multiple, independently regulated microdomains that control distinct functions by possessing unique effectors, targets, and means of regulating the concentration of the second messenger in cells [220]. Consequently, the effects of cAMP being easily diffused in cells are tightly controlled temporally and spatially. This notion may rationalize the findings that AC activities in higher plants are generally embedded in complex multidomain proteins [35], in addition to the tissue-specific expression of a few AC activity genes such as *PSiP* [31]. Obviously, these mechanisms confer the specific and high-efficiency regulatory roles of cAMP signaling in plants. In the present study, transgenic overexpression of the soluble AC may cause an overall disruption of cAMP signaling events that naturally take place during plant growth and development, which favors explaining the negative effects as described above. Plants are sessile organisms that need to continually adapt and modulate their rate of growth and development in accordance with the ever-changing environment, a phenomenon referred to as plasticity that involves a well-coordinated interaction between different signaling pathways, the spatiotemporal involvement of phytohormones, and cues from the environment [221]. Cellular cAMP levels in plants represent the pooled outcome of all AC (and PDE) activities expressed by different genes of diverse biological functions, which may in turn calibrate these signaling activities by feedback regulation. Our results supported that plants operate the well-established mechanisms of cAMP signaling in animals, which involve a complex network including key signaling pathways such as hormones, MAPK, lipids, Ca^2+^, sugar, and others. We envisage therefore that cAMP may act as integrator of various signals and function to coordinate systemic responses by forming an intricate and ingenious signaling system in plant plasticity.

## Figures and Tables

**Table 1:** Selected cAMP responsive genes (CRGs) in diverse biological processes.

Biological Processes	CRGs	
Phytohormone biosynthesis and homeostasis	PIN6 (AT1G77110), PID (AT2G34650), JAR1 (AT2G46370), DND1 (AT2G44810), AOC1 (AT3G25760), AOC2 (AT3G25770), GA1 (AT4G02780), CPS (AT4G02780), ACS8 (AT4G37770), LOG7 (AT5G06300), SMT3 (AT1G76090), CYP702A3 (AT4G15310), HXXXD-type acyl-transferase family protein (AT2G40230)	
mRNA degradation	SOV (AT1G77680), CAF1-5 (AT1G61470), TSN2 (AT5G61780), BRN2 (AT1G03457)	
Protein synthesis	LOS1 (AT1G56070)	
Proteasomal degradation	PUB29 (AT3G18710), PUB45 (AT1G27910), RING/U-box superfamily protein (AT5G47610), SGR9 (AT5G02750), SNIPER1 (AT1G14200), EBF2 (AT5G25350), PP2-A14 (AT5G52120), KMD2 (AT1G15670), KMD4 (AT3G59940), F-box/kelch-repeat protein (AT1G14330), HUP6 (AT3G27220), BTB/POZ domain-containing protein (AT2G30600)	
Ion transport and cell signaling		
Ca^2+^	AtCNGC2 (AT5G15410), CAX7 (AT5G17860), CSC1-like proteins (AT1G10090; AT1G62320)	
K^+^	AtKUP7	
Nitrate	NPF5.12 (AT1G72140), NPF7.2/NRT1.8 (AT4G21680), NRT2.6 (AT3G45060), NPF2.7/NAXT1(AT3G45650), CLC-b (AT3G27170)	
Sugar	SWEET16 (AT3G16690), HKL1 (AT1G50460), PMT6/PLT6 (AT4G36670)	
Lipid	MIOX2 (AT2G19800), ITPK3 (AT4G08170), SFH14 (AT5G56160)	
Light	BG1 (AT5G12050), ERD7 (AT2G17840), KNAT4 (AT5G11060), DFL2 (AT4G03400), PKS1 (AT2G02950), PKS2 (AT1G14280), BRN2 (AT1G03457), TEM1 (AT1G25560), BBX30 (AT4G15248)	
CO_2_/HCO_3_^−^/pH sensing	βCA3 (AT1G23730), SLAH3 (AT5G24030)	
Cell cycle	CDKG1 (AT5G63370)	
Secondary metabolism	AtGES (AT1G61120), CYP82G1 (AT3G25180), TAT3 (AT2G24850), DHS1 (AT4G39980), Tryptophan synthase (AT5G28237)	
Cell wall assembly and remodeling	XTH16 (AT3G23730), XTH23 (AT4G25810), XTH33 (AT1G10550), CSLA3 (AT1G23480), LRX2 (AT1G62440), AtGH9C2 (AT1G64390), KOR2 (AT1G65610), EXPA12 (AT3G15370), EXPA15 (AT2G03090), GALS1 (AT2G33570), FLA13 (AT5G44130), Exostosin family protein (AT4G32790), PME20 (AT2G47550), PMEI7 (AT4G25260), PMEI11 (AT3G47380), PMEI13/MMI9.1 (AT5G62360), MMI9.18 (AT5G62350), PER62 (AT5G39580), MAP70.5 (AT4G17220), EARLI1-like lipid transfer protein 3 (AT4G12500), Cell wall integrity/stress response component-like protein (AT4G39840), OFUT39 (AT5G65470), EXO70D1 (AT1G72470)	
Mitochondria-associated	PDC1 (AT4G33070), QCR7-2 (AT5G25450), PCMP-E34 (AT1G28690), PCMP-H43 (AT3G12770), POX1 (AT3G30775), POX2 (AT5G38710), DFR1 (AT5G17460), APC1 (AT5G61810), PUMP4 (AT4G24570)	
Chloroplasts-associated	MSL3 (AT1G58200)	

**Table 2:** cAMP responsive genes (CRGs) with functions of transcription factor activity.

GO Term	Up-Regulated CRGs	Down-Regulated CRGs
Transcription Factors (TF family) *		
GO:0003700~transcription factor activity andGO:0006355~regulation of transcription	AT1G19210 (ERF), AT1G74930 (ERF), AT4G25470 (ERF), AT4G25490 (ERF), AT5G21960 (ERF), AT5G47230 (ERF), AT5G51190 (ERF), AT5G51990 (ERF), AT5G61600 (ERF), AT2G38470 (WRKY), AT2G46400 (WRKY), AT4G23810 (WRKY), AT5G03150 (C2H2), AT5G67450 (C2H2), AT2G40140 (C3H), AT5G53210 (bHLH), AT5G11060 (TALE), AT1G46480 (WOX), AT3G10590 (MYB-related), AT5G66350 (SRS), AT2G17040 (NAC), AT3G21150 (C2C2 ^$^), AT4G14465 (AT-hook ^$^)	AT1G22810 (ERF), AT1G43160 (ERF), AT1G72360 (ERF), AT4G06746 (ERF), AT4G32800 (ERF), AT5G25810 (ERF), AT5G53290 (ERF), AT5G64750 (ERF),AT5G67190 (ERF), AT1G12540 (bHLH), AT1G18400 (bHLH), AT1G62975 (bHLH), AT1G73830 (bHLH), AT2G22770 (bHLH), AT4G29930 (bHLH), AT1G18710 (MYB), AT1G57560 (MYB), AT4G05100 (MYB), AT5G56840 (MYB-related), AT5G58900 (MYB-related), AT1G10480 (C2H2), AT2G41940 (C2H2), AT3G53600 (C2H2), AT5G44260 (C3H), AT4G16780 (HD-ZIP), AT4G17460 (HD-ZIP), AT4G37790 (HD-ZIP), AT3G01080 (WRKY), AT4G23550 (WRKY), AT2G38250 (Trihelix), AT5G01380 (Trihelix), AT1G25560 (RAV), AT3G25730 (RAV)AT1G14920 (GRAS), AT3G50650 (GRAS), AT3G02150 (TCP), AT2G28550 (AP2), AT1G69570 (Dof), AT5G49700 (AT-hook ^$^)
Regulation of Transcription (gene description)		
GO:0006355~regulation of transcription	AT1G61470 (CCR4-ASSOCIATED FACTOR 1E, CAF1E), AT2G40130 (SMAX1-LIKE 8, SMXL8), AT3G04930 (DNA-binding storekeeper protein-related transcriptional regulator), AT5G36740 (Acyl-CoA N-acyltransferase with RING/FYVE/PHD-type zinc finger), AT5G57180 (CHLOROPLAST IMPORT APPARATUS 2, CIA2)	

* Transcription factor (TF) family indicated in parenthesis was determined following the PlantTFDB v5.0 database [88], except those marked by symbol “$”, which were included in the RARTF database [87]. Gene Ontology (GO) annotation of CRGs (Supplementary Data S1) was performed using DAVID [85].

## Data Availability

All relevant data can be found within the manuscript and its supporting materials. The datasets generated and analyzed during the current study are available from the corresponding author upon reasonable request.

## Supplementary Materials

The following are available online at https://www.mdpi.com/article/10.3390/biom11050688/s1, Figure S1. Quantitative RT-PCR detection of cAMP-responsive genes (CRGs) in independent *AC* transgenic lines and wild type plants. Figure S2. Phenotypic characterization of cellular cAMP elevation in plants grown under different treatment regimes. Table S1. Primers used in this study. Table S2. List of cAMP-responsive gene sets. Table S3. WGCNA co-expression modules and correlation with cellular cAMP elevation. Table S4. Hub genes and CRGs in the depicted co-expression networks of WGCNA modules. Data S1. List of CRGs with FPKMs. Data S2. Functional annotation of CRGs. Data S3. Functional annotation of up- and down-regulated CRGs. Data S4. Biological pathways of CRGs. Data S5. Identification of cAMP-responsive gene sets by GSEA. Data S6. Functional annotation of WGCNA co-expression modules. Data S7. Top-ranking 10% genes with highest K_ME_ within WGCNA co-expression modules and their FPKMs.
