# Supplementary material for: Molecular Targets and Biological Functions of cAMP Signaling in Arabidopsis
Source: Biomolecules. 2021 May 3;11(5):688. doi: 10.3390/biom11050688 (PMC8147800; doi:10.3390/biom11050688)
Supplement: Supplementary file 1 [file biomolecules-11-00688-s001.zip › Supplementary Figure S1.pdf]

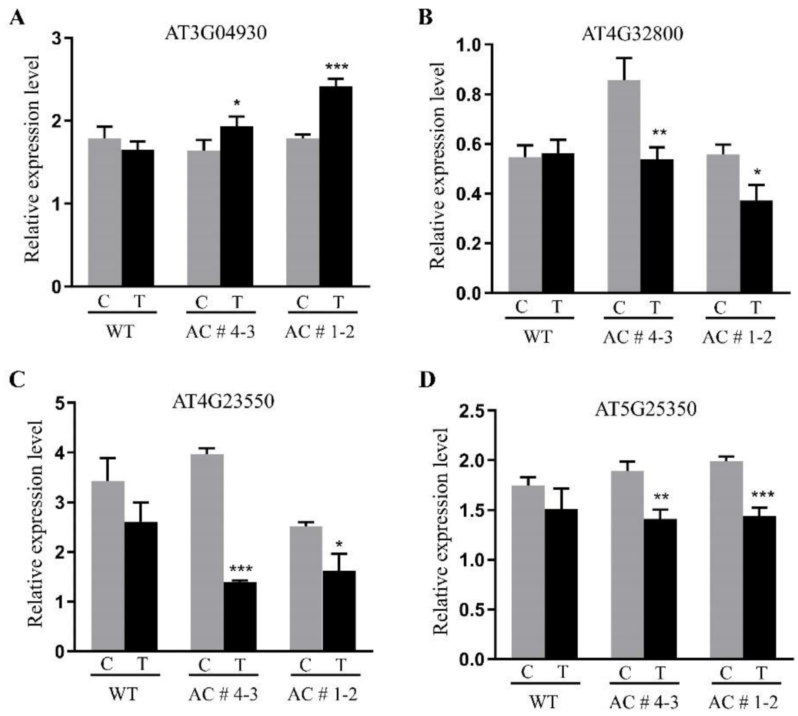

Supplementary Figure S1. Quantitative RT-PCR detection of cAMP-responsive genes (CRGs) in independent *AC* transgenic lines and wild type plants. About two-week-old *AC* transgenic plants (transgenic lines AC #4-3 and AC #1-2) and wild type (WT) plants were thoroughly sprayed with 30 µM dexamethasone (T) or mock control (C), and then covered overnight using a transparent plastic dome. Shoot tissue samples were collected 24 h after spray and used for total RNA isolation. Quantitative RT-PCR was performed to detect relative expression of CRGs using the $2^{-\Delta\Delta Cq}$ method. β-tubulin (*AtTUB4*) was used as an internal control. (**A**) AT3G04930. (**B**) AT4G32800. (**C**) AT4G23550. (**D**) AT5G25350. Data are Ave ± SD (n = 3), two-tailed Student's *t*-test $*p < 0.05$, $**p < 0.01$, $***p < 0.001$.
