# Supplementary material for: Molecular Targets and Biological Functions of cAMP Signaling in Arabidopsis
Source: Biomolecules. 2021 May 3;11(5):688. doi: 10.3390/biom11050688 (PMC8147800; doi:10.3390/biom11050688)
Supplement: Supplementary file 1 [file biomolecules-11-00688-s001.zip › Supplementary Figure S2.pdf]

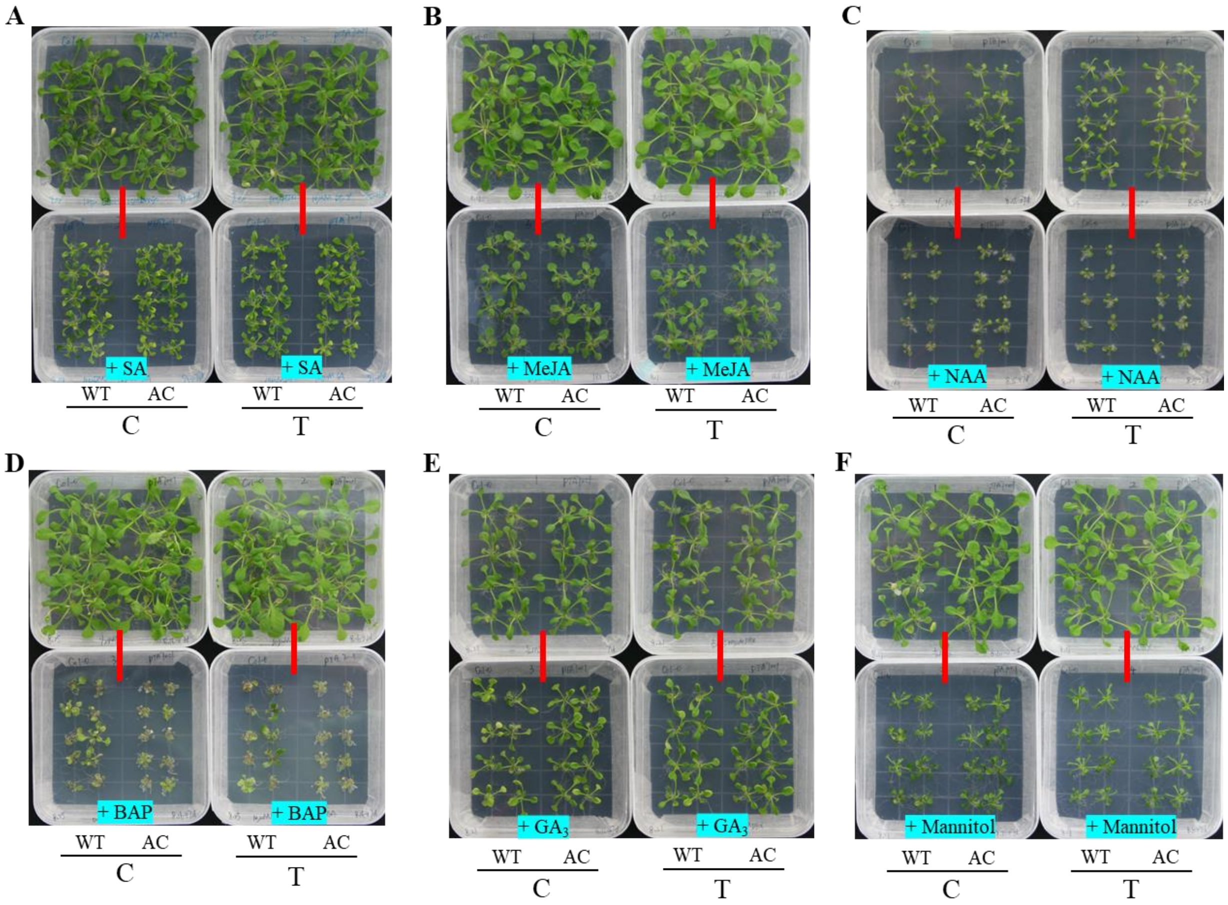

Supplementary Figure S2. Phenotypic characterization of cellular cAMP elevation in plants grown under different treatment regimes. (**A**) 100 µM SA (salicylic acid), photograph taken 10 days after treatment. (**B**) 50 µM MeJA (methyl jasmonate), photograph taken 12 days after treatment. (**C**) 0.1 µM NAA (1-naphthaleneacetic acid), photograph taken 4 days after treatment. (**D**) 0.1 µM BAP (6-benzylaminopurine), photograph taken 14 days after treatment. (**E**) 10 µM GA_3 (gibberellic acid), photograph taken 7 days after treatment. (**F**) 100 mM mannitol, photograph taken 10 days after treatment. In (A) to (F), seven-day-old *AC* transgenic seedlings and wild type (WT) Col-0 seedlings were transplanted onto 1/2MS basal salt growth medium containing 10 µM dexamethasone (T; right column panels) or mock control (C; left column panels) with the addition of indicated treatments (bottom row panels) or mock control (upper row panels).
