# Supplementary material for: Molecular Targets and Biological Functions of cAMP Signaling in Arabidopsis
Source: Biomolecules. 2021 May 3;11(5):688. doi: 10.3390/biom11050688 (PMC8147800; doi:10.3390/biom11050688)
Supplement: Supplementary file 1 [file biomolecules-11-00688-s001.zip › Supplementary Table S1.pdf]

Supplementary Table S1. Primers used in this study

Gene ID	Sequence (5'→3')	Size (bp)
At5G09400<br>(*AtKUP7*)	Forward: <u>CACC</u> ATG GCG GAG GAA AGC AGT AT<br>Reverse: <u>TTA</u> TTT CCT CCC AAC GGT CAA G	319 (RNA)<br>882 (gDNA)
AC transgene	Forward: ATG GCG GAG GAA AGC AGT AT<br>Reverse: TTT CCT CCC AAC GGT CAA G	312
At5G44340<br>(*AtTUB4*)	Forward: TGT TTC GTT CAT GTG TGT TCG TT<br>Reverse: ACA CGC AAA AGT TTA ACA AAT CCA	74
AT1G14920	Forward: GAC GAC CTG GTG CGA TCG AT<br>Reverse: GGT ACA CCT TCC AAC GAG TCA	169
AT1G61120	Forward: TGT CCA TCT TCG CAA CAT CTG G<br>Reverse: TGT GTG CTG CGA TTG AGA TCA TTC	131
AT2G38310	Forward: CTA GCT CCA CCG AGA GAC TC<br>Reverse: AAC GAC GTA AGA CTC GAC AAC GA	158
AT2G38470	Forward: TCT TGA GAT ACC AAA GTT CAG ATC<br>Reverse: TGG TTG GAG AAG CTA GAA CGT TA	152
AT2G46370	Forward: CTG CCA TTT CCT TAA GCT CTG GA<br>Reverse: ATG TAC TGC TTG CTG CTG AAG ATA	181
AT3G04930	Forward: GAT AAA GTC GAA ACT TCA GCT GGA T<br>Reverse: AGA CTG ATC ATG AGG GCT CTT GA	142
AT3G09870	Forward: GGA AGA GGG TAA TGC TGC ATC A<br>Reverse: CCC TTA ACC GCA ATC ACT GCT A	75
AT4G03400	Forward: GTT CTA ACC ACT TTC ACA GGT CTT<br>Reverse: AGA AGC TGA GAC GCC TTG TCA	185
AT4G23550	Forward: CGA ACC GGA GAT GGA GAC AAG T<br>Reverse: CCG AGC TTG TGA GGA TCG TTT G	95
AT4G27260	Forward: CTC TTA CAC GCT CAT ACC ATC CA<br>Reverse: TCT GCA AAG CCC GGC GTA AG	193
AT4G32800	Forward: AAA GTT ACC GAG TCT AGG AAC GA<br>Reverse: CCA CCG AGG CAT GTA CAC CA	91
AT5G25350	Forward: GGT TGG GAC GTC GAG TCG T<br>Reverse: CAA CAT CAG TCA CCT TAC TCT CA	80
AT5G47230	Forward: CTC TTG AAG TTG GGA AGT GGA A<br>Reverse: ACC GTT AAC GTC AAC GCT CTG T	128
AT3G18780<br>(*AtACT2*)	Forward: CTT GCA CCA AGC AGC ATG AA<br>Reverse: CCG ATC CAG ACA CTG TAC TTC CTT	68
Vd991-ITS	Forward: CGG TGT TGG GGA TCT ACG TC<br>Reverse: CGG GCT TGT AGG GGG TTT AG	129

Note: the underline indicates nucleotides added for cloning purpose.
