# Supplementary material for: Molecular Targets and Biological Functions of cAMP Signaling in Arabidopsis
Source: Biomolecules. 2021 May 3;11(5):688. doi: 10.3390/biom11050688 (PMC8147800; doi:10.3390/biom11050688)
Supplement: Supplementary file 1 [file biomolecules-11-00688-s001.zip › Supplementary Table S2.pdf]

Supplementary Table S2. List of cAMP-responsive gene sets

Gene Set	Size	Core Subset	NES	*P*-value	FDR
**GO term-based**					
Intracellular signal transduction	489	102	-1.48	1.2E-03	0.21
Apoplast	398	87	-1.56	1.2E-03	0.21
Response to oxidative stress	386	76	-1.44	1.2E-03	0.21
Glycosyl compound metabolism	207	76	-1.86	1.4E-03	0.21
Response to wounding	185	62	-1.77	1.4E-03	0.21
Secondary metabolic process	381	123	-1.42	2.5E-03	0.21
Sulfur compound metabolic process	340	122	-1.46	2.6E-03	0.21
Ethylene-activated signaling pathway	153	46	-1.73	2.8E-03	0.21
Phosphorelay signal transduction system	170	47	-1.68	2.8E-03	0.21
Intracellular	142	44	-1.72	2.9E-03	0.21
Response to chitin	113	42	1.97	3.0E-03	0.21
S-glycoside metabolic process	102	53	-1.76	3.0E-03	0.21
Pattern specification process	153	55	1.88	3.4E-03	0.21
Response to organonitrogen compound	209	48	1.67	3.7E-03	0.21
Cell division	248	98	1.61	4.1E-03	0.21
Response to nitrogen compound	254	53	1.57	4.1E-03	0.21
Transferase activity, transferring alkyl or aryl groups	133	52	-1.71	4.4E-03	0.21
Chemical homeostasis	322	65	1.71	4.7E-03	0.21
Molecular function regulator	382	77	-1.40	4.9E-03	0.21
Enzyme regulator activity	301	78	-1.47	5.1E-03	0.21
ATPase activity	361	166	1.36	5.2E-03	0.21
Microtubule motor activity	60	42	1.72	5.2E-03	0.21
Response to drug	391	68	1.45	5.3E-03	0.21
Kinesin complex	58	42	1.81	5.3E-03	0.21
Abscisic acid-activated signaling pathway	228	52	-1.55	5.4E-03	0.21
Glucosyltransferase activity	179	46	-1.61	5.6E-03	0.21
Lipid catabolic process	196	43	-1.55	5.6E-03	0.21
Homeostatic process	494	82	1.50	5.6E-03	0.21
Microtubule associated complex	87	58	1.82	5.7E-03	0.21
Response to jasmonic acid	151	44	-1.66	5.7E-03	0.21
Transferase activity, transferring glycosyl groups	472	111	-1.38	5.9E-03	0.21
Tissue development	126	49	1.74	6.1E-03	0.21
Anatomical structure formation involved in morphogenesis	141	55	1.64	6.5E-03	0.21
Copper ion binding	183	50	-1.57	6.9E-03	0.21
Metal ion transmembrane transporter activity	179	45	1.68	7.0E-03	0.21
Auxin-activated signaling pathway	181	58	1.63	7.2E-03	0.21
Metal ion transport	181	43	1.58	7.2E-03	0.21
Ion homeostasis	225	44	1.54	7.6E-03	0.21
Hormone metabolic process	186	57	-1.49	1.1E-02	0.23
**KEGG pathway-based**					
Phenylalanine, tyrosine and tryptophan biosynthesis	52	28	-1.80	1.6E-03	0.13
α-Linolenic acid metabolism	34	10	-1.91	1.6E-03	0.13
Phosphatidylinositol signaling system	58	23	-1.68	3.0E-03	0.13
Inositol phosphate metabolism	61	20	-1.68	3.1E-03	0.13
Estrogen signaling pathway	26	11	-1.78	3.3E-03	0.13

Note: Gene sets were determined using GSEA (Gene Set Enrichment Analysis) with the false discovery rate (FDR) < 0.25 of normalized enrichment score (NES). Size, number of genes in the gene set. Core Subset, number of genes in the leading-edge subset that contribute most to the enrichment result; for a list of core genes, see Supplementary Data S5.
