# Supplementary material for: Molecular Targets and Biological Functions of cAMP Signaling in Arabidopsis
Source: Biomolecules. 2021 May 3;11(5):688. doi: 10.3390/biom11050688 (PMC8147800; doi:10.3390/biom11050688)
Supplement: Supplementary file 1 [file biomolecules-11-00688-s001.zip › Supplementary Table S3.pdf]

Supplementary Table S3. WGCNA co-expression modules and correlation with cellular cAMP elevation

Module name	Size	ME vs. Trait		GS vs. MM		CRGs	Hubs
	(n)	R	*p*-value	R	*p*-value	(n)	(n)
**Darkorange2**	3183	**0.88**	**0.02**	0.59	<1e-200	88	3
**Cornflowerblue**	786	**0.88**	**0.02**	0.64	8.7e-92	31	3
**Peachpuff2**	140	**0.88**	**0.02**	0.54	5.8e-12	2	3
Forestgreen	782	0.68	0.1	0.24	0.1e-10	1	3
Lightpink3	357	0.68	0.1	0.17	1.3e-03	0	3
Royalblue3	469	0.68	0.1	-0.058	0.21	0	3
Steelblue	663	0.68	0.1	0.53	2.7e-49	2	3
Dodgerblue3	1096	0.49	0.3	0.059	0.051	5	2
Goldenrod3	345	0.49	0.3	-0.018	0.74	0	3
Lemonchiffon4	604	0.49	0.3	-0.3	5e-14	0	3
Orangered2	320	0.49	0.3	0.0092	0.87	0	3
Burlywood	1260	0.29	0.6	0.03	0.29	1	3
Chocolate3	318	0.29	0.6	-0.065	0.25	0	3
Deeppink	246	0.29	0.6	0.23	2.7e-4	0	3
Deepskyblue3	452	0.29	0.6	0.16	6.4e-4	0	3
Green	897	0.29	0.6	0.22	2.7e-11	0	1
Red4	184	0.29	0.6	0.12	0.1	0	3
Bisque4	1677	0.098	0.9	-0.45	2.2e-84	0	3
Brown4	2915	0.098	0.9	-0.36	6.5e-90	0	3
Chartreuse4	890	0.098	0.9	0.17	3.4e-07	1	2
Cornsilk	285	0.098	0.9	-0.11	0.064	0	3
Salmon4	249	0.098	0.9	-0.13	0.04	0	3
Dodgeblue2	662	-0.098	0.9	-0.47	1.1e-37	0	3
Lightseagreen	158	-0.098	0.9	-0.14	0.079	0	3
Lightskyblue3	122	-0.098	0.9	-0.33	2.1e-04	0	3
Mistyrose4	2326	-0.098	0.9	0.049	0.018	1	1
Skyblue1	869	-0.098	0.9	0.2	2.7e-09	1	2
Cyan1	612	-0.29	0.6	0.31	4.2e-15	2	3
Cyan3	182	-0.29	0.6	-0.05	0.5	0	3
Moccasin	144	-0.49	0.3	0.24	3.8e-3	1	3
Antiquewhite	233	-0.68	0.1	0.49	1.8e-15	2	3
Paleturquoise	734	-0.68	0.1	0.46	0.1e-38	4	3
**Orange**	171	**-0.88**	**0.02**	0.12	0.12	0	3
**Aquamarine1**	841	**-0.88**	**0.02**	0.055	0.11	1	3
**Chocolate1**	3119	**-0.88**	**0.02**	0.76	<1e-200	284	3

Note: WGCNA (Weighted Correlation Network Analysis) was performed to determine co-expression modules. The modules in bold indicate their significant relationship with cellular cAMP elevation by the threshold $p < 0.05$ of correlation coefficient (R). Size, number of genes in the module; CRGs, number of cAMP-responsive genes included in the module; Hubs, number of hub genes that were defined with more than 4 connections in the constructed network of top 10% genes with highest K_{ME} in the module, wherein K_{ME} represents eigengene connectivity measured for each gene by correlating its gene expression profile with the module eigengene (ME) which is defined as the first principal component of gene expression values and represents the gene expression profiles in the module. ME vs. Trait, the correlation between ME and trait (i.e. induction of cAMP elevation); GS vs. MM, the correlation between module membership (MM, also known as K_{ME}) and gene significance (GS) which is defined as the correlation of gene expression profiles with the trait.
