# Supplementary material for: Molecular Targets and Biological Functions of cAMP Signaling in Arabidopsis
Source: Biomolecules. 2021 May 3;11(5):688. doi: 10.3390/biom11050688 (PMC8147800; doi:10.3390/biom11050688)
Supplement: Supplementary file 1 [file biomolecules-11-00688-s001.zip › Supplementary Table S4.pdf]

Supplementary Table S4. Hub genes and CRGs in the depicted co-expression networks of WGCNA modules

Module & Gene ID	Description	K_{ME}	Hubs (connections)
**Chocolate1**			
AT5G58900	Homeodomain-like transcriptional regulator	1.00	Y (61)
AT1G06410	Trehalose-phosphatase/synthase 7 (TPS7)	1.00	Y (99)
AT5G07920	Diacylglycerol kinase1(DGK1)	1.00	Y (43)
CRGs	AT1G12450; AT1G14330; AT1G16370; AT1G22360; AT1G27910; AT1G44350; AT1G51090; AT1G72470; AT1G72510; AT1G75310; AT1G76160; AT2G17840; AT2G19780; AT2G22770; AT2G31010; AT2G34770; AT2G38310; AT2G39130; AT2G40130; AT2G40140; AT4G19530; AT5G03150; AT5G24030; AT5G25350; AT5G38710; AT5G40380; AT5G44050; AT5G52120; AT5G56160; AT5G58680; AT5G58900; AT5G64750; AT5G65470; AT5G65630	>0.98	
**Darkorange2**			
AT2G21050	LIKE AUXIN RESISTANT 2 (LAX2)	1.00	Y (5)
AT1G02270	Calcium-binding endonuclease/exonuclease/phosphatase family	1.00	Y (108)
AT5G51130	S-adenosyl-L-methionine-dependent methyltransferases superfamily protein	1.00	Y (89)
CRGs	AT1G61120; AT2G03090; AT2G15020; AT2G15760; AT2G28550; AT2G30930; AT2G44670; AT3G25870; AT4G15233; AT4G27260; AT4G29930; AT4G37530; AT5G12340; AT5G58670; AT5G63370	>0.96	
**Cornflowerblue**			
AT1G66090	Disease resistance protein (TIR-NBS class)	1.00	Y (63)
AT5G09400	K^+ uptake permease 7 (KUP7)	1.00	Y (64)
AT1G19300	Nucleotide-diphospho-sugar transferases superfamily protein (PARVUS)	1.00	Y (73)
CRGs	AT1G12540; AT1G72920; AT2G17040; AT2G28120; AT2G32510; AT2G46370; AT4G03390; AT4G32350; AT5G09400; AT5G10410; AT5G59580; AT5G62360; AT5G63450; AT5G67420	>0.98	

Note: WGCNA (Weighted Correlation Network Analysis) was performed to determine co-expression modules. The top-ranking 10% of genes with highest K_{ME} in the module (Supplementary Data S7) were used to depict a subnetwork, and hub genes (indicated by "Y") were determined as those with at least 4 depicted connections in the network (Figure 7c-e). K_{ME} represents eigengene connectivity measured for each gene by correlating its gene expression profile with the module eigengene (ME) which is defined as the first principal component of gene expression values and represents the gene expression profiles in the module. CRGs, cAMP-responsive genes. The underline of Gene ID indicates that the hub genes belong to the CRGs.
